# Prediction of KPC-producing *Klebsiella pneumoniae* by MALDI-TOF MS, ensemble learning, and spectral peak annotation

**DOI:** 10.1128/jcm.01466-25

**Published:** 2026-03-30

**Authors:** David Rodriguez-Temporal, Mark Gutiérrez-Pareja, Garrett G. Gordy, Ellen M. Nahkala, Belén Rodríguez-Sánchez, Robin Patel

**Affiliations:** 1 Division of Clinical Microbiology, Department of Laboratory Medicine and Pathology, Mayo Clinic 6915, Rochester, Minnesota, USA; 2 Clinical Microbiology and Infectious Diseases, Hospital General Universitario Gregorio Marañón, Instituto de Investigación Sanitaria Gregorio Marañón (IISGM), Madrid, Spain; 3 Division of Public Health, Infectious Diseases, and Occupational Medicine, Mayo Clinic 6915, Rochester, Minnesota, USA

**Keywords:** carbapenemase, machine learning, KPC, MALDI-TOF, *Klebsiella pneumoniae*

## Abstract

The increasing prevalence of carbapenem-resistant *Klebsiella pneumoniae* represents a serious global health challenge. Rapid and accurate detection methods are essential to inform early and correct use of antimicrobial therapy to idealize patient outcomes and limit the spread of resistant isolates. Matrix-assisted laser desorption/ionization time-of-flight mass spectrometry (MALDI-TOF MS) has been evaluated for resistance detection, but its clinical application remains limited. In this study, MALDI-TOF MS was integrated with machine learning (ML) to improve detection of *Klebsiella pneumoniae* carbapenemase (KPC) among *K. pneumoniae* isolates harboring KPC carbapenemase. Ensemble learning strategies were applied to 435 clinical isolates from different geographic locations (Spain, United States, South America, and Asia), with validation performed using external data sets. By constructing two peak matrices and applying four ML algorithms, as well as combinations of two and three in ensemble models, 92 different classifiers were tested. Ensemble combinations increased the specificity of classifiers to over 95%, while sensitivity reached 72%, being significantly higher than that of the MALDI Biotyper KPC module. Low sensitivity may be affected by technical variability during spectral acquisition. The first annotated MALDI-TOF MS spectrum for *K. pneumoniae* by *in silico* prediction of protein masses was developed to enable peak identification, including peaks potentially related to antimicrobial resistance. Overall, the results of this study show that combining MALDI-TOF MS with ensemble learning can enhance KPC detection performance.

**IMPORTANCE:**

Rapid and accurate detection of *Klebsiella pneumoniae* carbapenemase (KPC)-producing *Klebsiella pneumoniae* informs early and correct use of antimicrobial therapy to idealize patient outcomes and limit the spread of antimicrobial resistance. In this study, matrix-assisted laser desorption/ionization time-of-flight mass spectrometry (MALDI-TOF MS) and ensemble machine learning were employed to detect KPC-producing isolates, using diverse isolate collections, obtaining excellent specificity. An annotated *K. pneumoniae* MALDI-TOF MS profile is provided with peak annotation, providing a resource for peak analysis.

## INTRODUCTION

The rise of antimicrobial resistance (AMR) represents a threat to global health, with an estimated 1.27 million deaths globally directly attributable to antimicrobial-resistant infections in 2019 (1). According to the World Health Organization, carbapenem-resistant Enterobacterales are among the highest-priority pathogens for research and development of strategies aimed at controlling and preventing the spread of resistance (2). A common clinically relevant member of this group is *Klebsiella pneumoniae*, particularly isolates producing class A carbapenemases, such as *K. pneumoniae* carbapenemase (KPC) (3). The increasing prevalence of KPC-producing *K. pneumoniae* compromises treatment options, leading to poor clinical outcomes and economic burden. Rapid and accurate diagnostic tools are needed to enable timely, targeted administration of antimicrobial therapy, thereby optimizing patient outcomes and helping to mitigate the spread of resistance (4).

Conventional methods for detecting AMR, such as disk diffusion and broth microdilution, are time-consuming and typically require an additional day, if not more, following bacterial isolation. As a result, efforts have been directed toward leveraging advanced technologies to accelerate AMR detection. Matrix-assisted laser desorption/ionization time-of-flight mass spectrometry (MALDI-TOF MS) has transformed bacterial identification and is now the standard method for the identification of most bacteria grown in culture. This type of testing yields rapid results and can be performed as soon as bacteria are recovered in culture. This technology has not been clinically applied for the detection of AMR on a routine basis; although if reliable resistance profiling were possible, the speed and cost-effectiveness of such an approach would make it useful in clinical practice (5). Unfortunately, resistance detection by MALDI-TOF MS remains technically challenging (6).

In recent years, integration of machine learning (ML) algorithms with MALDI-TOF MS data has emerged as a promising strategy for AMR detection (7). This approach uses latent information embedded within mass spectra that may be associated with underlying resistance. ML models can recognize complex patterns and subtle spectral variations within the high-dimensional data generated by MALDI-TOF MS; such patterns may be imperceptible through traditional peak-based analysis. Several preliminary studies have explored the classification of carbapenem-resistant vs carbapenem-susceptible *K. pneumoniae* isolates, reporting promising results (8–10). Most are limited by reliance on single-center collections and the absence of validation with isolates from other centers or geographic regions. Additionally, protein peaks proposed in the literature as putative biomarkers remain poorly characterized and have yet to be validated across diverse isolate collections to confirm accuracy.

Lau et al. proposed the 11,109 *m*/*z* peak, representing a protein of unknown function encoded by a gene adjacent to the *bla*_KPC_ Tn*4401* transposon on the pKpQIL and other related plasmids, as a potential biomarker for KPC-producing isolates (11). Excellent specificity of this peak for the detection of KPC-producing isolates has been shown, since the gene associated with this peak has only been found adjacent to *bla*_KPC_. However, *bla*_KPC_ may be found without the pKpQIL gene, resulting in low sensitivity (28%–99%) of this single 11,109 *m*/*z* peak as a diagnostic biomarker (12–16). This limitation underscores the need for more advanced strategies, such as the use of additional mass spectral peaks and/or the application of ML techniques to enhance the detection of KPC-producing isolates. The development of such integrated strategies holds potential to improve clinical microbiology workflows by quickly and easily informing optimal therapeutic options and strengthening antimicrobial stewardship.

Here, clinical *K. pneumoniae* isolates from different regions were used to develop ML algorithms for the detection of KPC-producing isolates. Ensemble learning, a methodology little explored in the context of microbial classification by MALDI-TOF MS (17), was used. In addition, a comprehensive *in silico* analysis, combining mass spectra with whole-genome sequence data, was performed, enabling the creation of the first annotated MALDI-TOF MS spectral data set. This integrated approach complements previous knowledge and offers new perspectives for future studies using microbial spectra to predict AMR.

## MATERIALS AND METHODS

### Bacterial isolates, culture, and identification

A total of 435 clinical isolates of *K. pneumoniae* were analyzed in this study, including KPC-producing (*n* = 237) and non-KPC-producing (*n* = 198) isolates (Table S1). The non-KPC group included both carbapenem-susceptible and carbapenem-resistant isolates, the latter with resistance mechanisms other than KPC production. Isolates were sourced from three collections: (i) 81 clinical isolates from Hospital General Universitario Gregorio Marañón, Madrid, Spain (HGM); (ii) 84 carbapenem-susceptible clinical isolates collected between January 2025 and March 2025 at Mayo Clinic, Rochester, MN (referred to as Mayo Clinic isolates); and (iii) 270 clinical Antibacterial Resistance Leadership Group Biorepository isolates, originating from across the United States, Colombia, Chile, Lebanon, and Singapore from the Consortium of Resistance Against Carbapenems in *Klebsiella* and other Enterobacterales (referred to as ARLG isolates) (18, 19). KPC in carbapenem-resistant isolates was assessed by the NG-Test Carba 5 (NG-Biotech, Guipry-Messac, France) for the HGM isolates, while for the ARLG isolates, it was assessed by WGS analysis.

*K. pneumoniae* isolates were cultured from frozen stocks onto Columbia sheep blood agar (bioMérieux, Marcy-l'Étoile, France) or 5% sheep blood trypticase soy agar (BD, Franklin Lakes, NJ, USA) and incubated for 24 h at 37°C. For MALDI-TOF MS analysis, the instrument was calibrated before each run with the Bacterial Test Standard (BTS, Bruker Daltonics, Bremen, Germany); bacterial colonies were processed using the on-plate extraction method by depositing 1 μL of 70% formic acid onto each spot and covering each with 1 μL HCCA matrix after drying (Bruker Daltonics). The HGM collection was analyzed using the MALDI Biotyper Smart system (Bruker Daltonics) and the Mayo Clinic and ARLG collections using the MALDI Biotyper Sirius system (Bruker Daltonics). For both analyses, disposable target plates (Bruker Daltonics) were used, and spectra were acquired in the range of 2,000–20,000 *m*/*z* using manufacturer-recommended settings for species identification. In addition, the MALDI Biotyper KPC detection module, which reports a KPC positive result when the single peak of 11,109 *m*/*z* is detected, was applied.

### Genomic characterization

Assembled genomes of ARLG isolates, which were previously published (18, 19), were obtained from GenBank. Multi-locus sequence typing (MLST) was performed using PubMLST schemes (https://github.com/tseemann/mlst), resistance genes were analyzed by Resfinder v3.2 (20), and capsule typing and virulence genes were assessed by Kleborate v2.3.2 (21). The genomic information is provided in Table S1.

### Spectral processing and machine learning models

**Fig 1:**
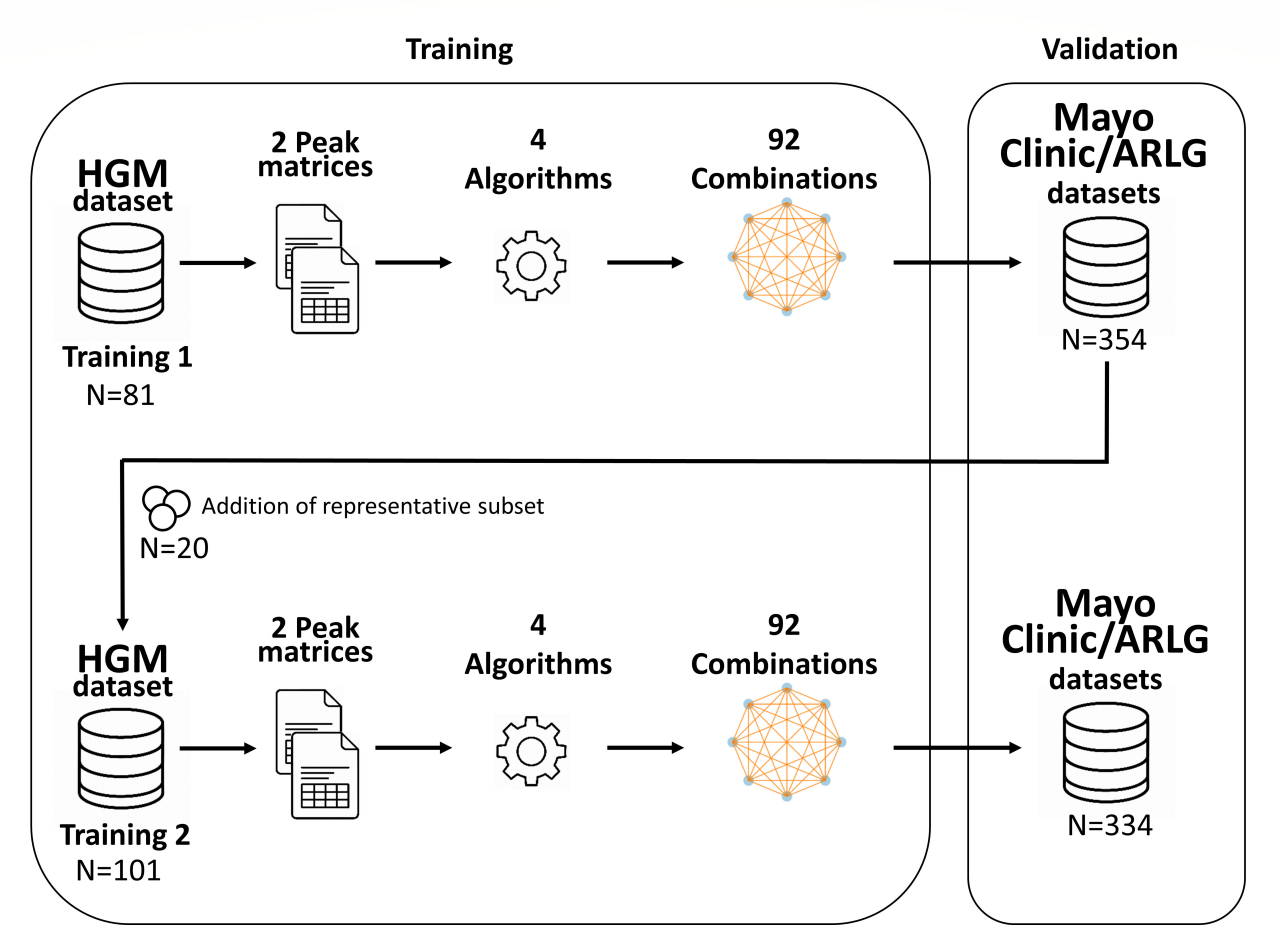
Schematic of the development of the training set and validation of the models used in this study.

MALDI-TOF MS spectra were processed using Clover MS Data Analysis Software (Clover MSDAS; Clover Biosoft, Granada, Spain). The preprocessing pipeline included smoothing (Savitzky-Golay filter with window length = 11, polynomial order = 3), baseline subtraction with a top-hat filter (threshold = 0.02), alignment (medium shift, constant tolerance = 2 *m*/*z*, and linear mass tolerance = 300 ppm), and normalization by total ion current. The HGM isolates (41 KPC and 40 non-KPC isolates) were used for the creation of the first training set (Training 1), with the Mayo Clinic and ARLG isolates used for external validation. To assess whether model performance could be enhanced through integration of heterogeneous data, a second training set (Training 2) was constructed by incorporating a representative subset of 20 isolates from the Mayo Clinic/ARLG collections (10 KPC and 10 non-KPC isolates) into the original training set, followed by re-validation with the remaining spectra (Fig. 1).

For model development, two distinct peak matrices were evaluated: (i) a full-spectrum peak matrix (F) and (ii) a reduced peak matrix consisting of the five most discriminative peaks (5P), selected based on area under the curve (AUC) values indicating relevance for KPC isolates. This peak-selection method is available in Clover MSDAS. Then, both peak matrices were used to train supervised machine learning algorithms, including partial least squares-discriminant analysis (PLS-DA), support vector machine (SVM), light-gradient boosting machine (LGBM), and random forest (RF). Model performance was assessed using 10-fold cross-validation.

To evaluate whether predictive performance could be improved through model integration, an ensemble learning framework was implemented by combining models in pairs and groups of three. Specifically, eight individual models (four algorithms for two peak matrices) were combined in all possible pairwise (*n* = 28) and triplet (*n* = 56) configurations, resulting in 92 evaluated models and ensemble combinations (Fig. 1). For each ensemble, a definitive prediction (KPC or non-KPC) was accepted only when all constituent models agreed. Sensitivity, specificity, positive predictive value (PPV), and negative predictive value (NPV) were calculated by R software (v4.4.2).

### Spectral annotation

To annotate *K. pneumoniae* mass spectra, they were processed using a recently described genomically predicted protein mass database (GMPsDB) (22). Although this database was created for identification, it enables the tentative assignment of mass spectral peaks to specific proteins based on *in silico*-predicted masses derived from whole-genome sequences. The database was downloaded from GitHub (https://github.com/ysekig/GPMsDB-tk) and implemented using Python (v3.13). After obtaining putative proteins from GMPsDB, considering known post-translational modifications (PTMs), such as the N-terminal methionine cleavage and the presence of signal peptides, they were evaluated by searching for corresponding genes within genomes of the ARLG isolates (to confirm their presence). Protein mass values and species-specific occurrences were confirmed using the Uniprot database. Ribosomal protein masses were also confirmed by the Ribopeaks database (23). All matched peaks were visually inspected in Clover MSDAS for confirmation and detection of double-charged ions. Finally, an interactive mass spectrum image with annotated proteins was created using Python.

### Statistical analysis

With Clover MSDAS, *t*-tests were applied to compare intensity mean values between peaks present in the protein spectra from KPC and non-KPC isolates. With R software, the following composite metrics were obtained for each model: Youden’s index, which measures the effectiveness of a test (values ranging from 0 to 1); balanced accuracy, which measures the mean between sensitivity and specificity and offers a general view of the model; and F1 score, which combines sensitivity and PPV and is useful when the aim is to minimize false-negative and false-positive results. For the analysis of statistical significance between the ensemble models and training groups, *t*-tests were used when the data were normally distributed, and Mann-Whitney tests were used when the data were not. A *P* value <0.05 was considered statistically significant.

## RESULTS

### Peak selection

**Fig 2:**
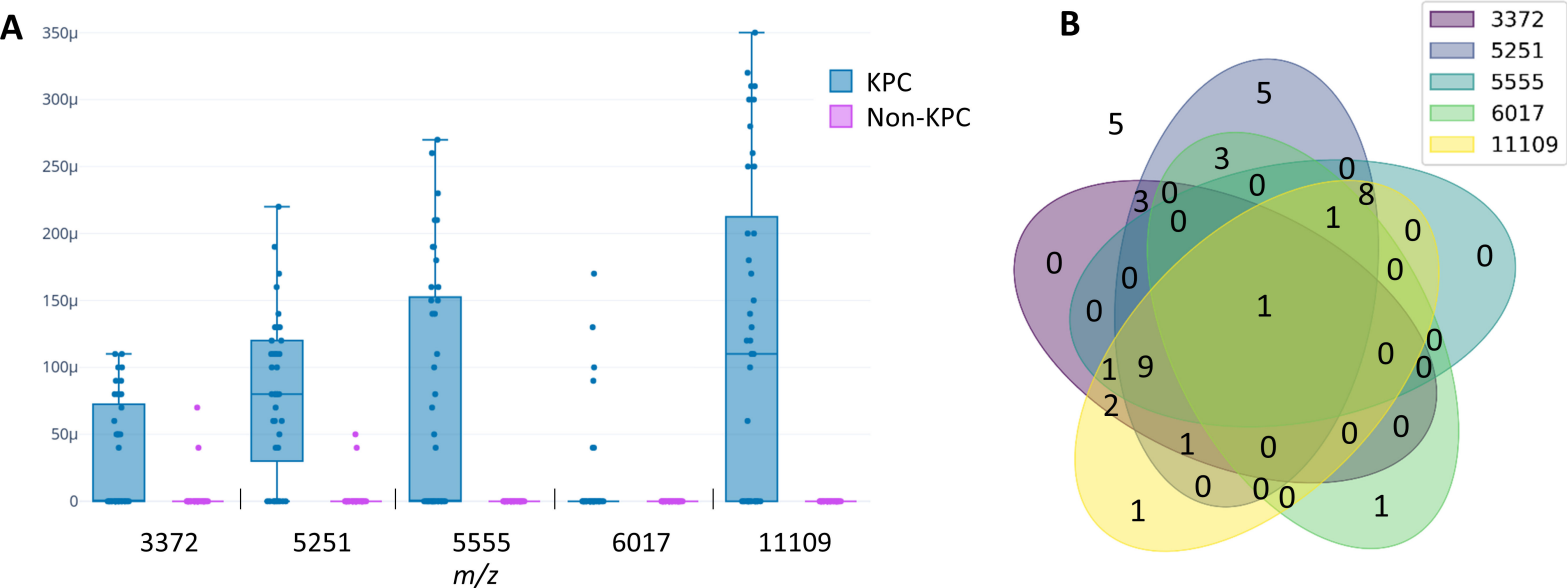
(**A**) Comparison of intensities of each peak between KPC and non-KPC isolates used in the first training set. (**B**) Venn diagram showing the number of isolates that share each peak among the first training set isolates.

The first training set (Training 1) consisted of two peak matrices: the full spectra and the reduced five-peak matrix. The five most discriminative peaks for KPC-producing isolate detection were 3,372 *m*/*z* (AUC = 0.67), 5,251 *m*/*z* (AUC = 0.87), 5,555 *m*/*z* (AUC = 0.74), 6,017 *m*/*z* (AUC = 0.56), and 11,109 *m*/*z* (AUC = 0.79). These five peaks yielded a statistically significant value by *t*-test (*P* < 0.05) by comparing mean intensities of KPC and non-KPC isolates (Fig. 2A). However, these peaks were not equally present in all KPC isolates (Fig. 2B), and 5/41 (12%) KPC isolates in the training set did not show any of the five selected peaks.

### Model validation from Training 1

**Fig 3:**
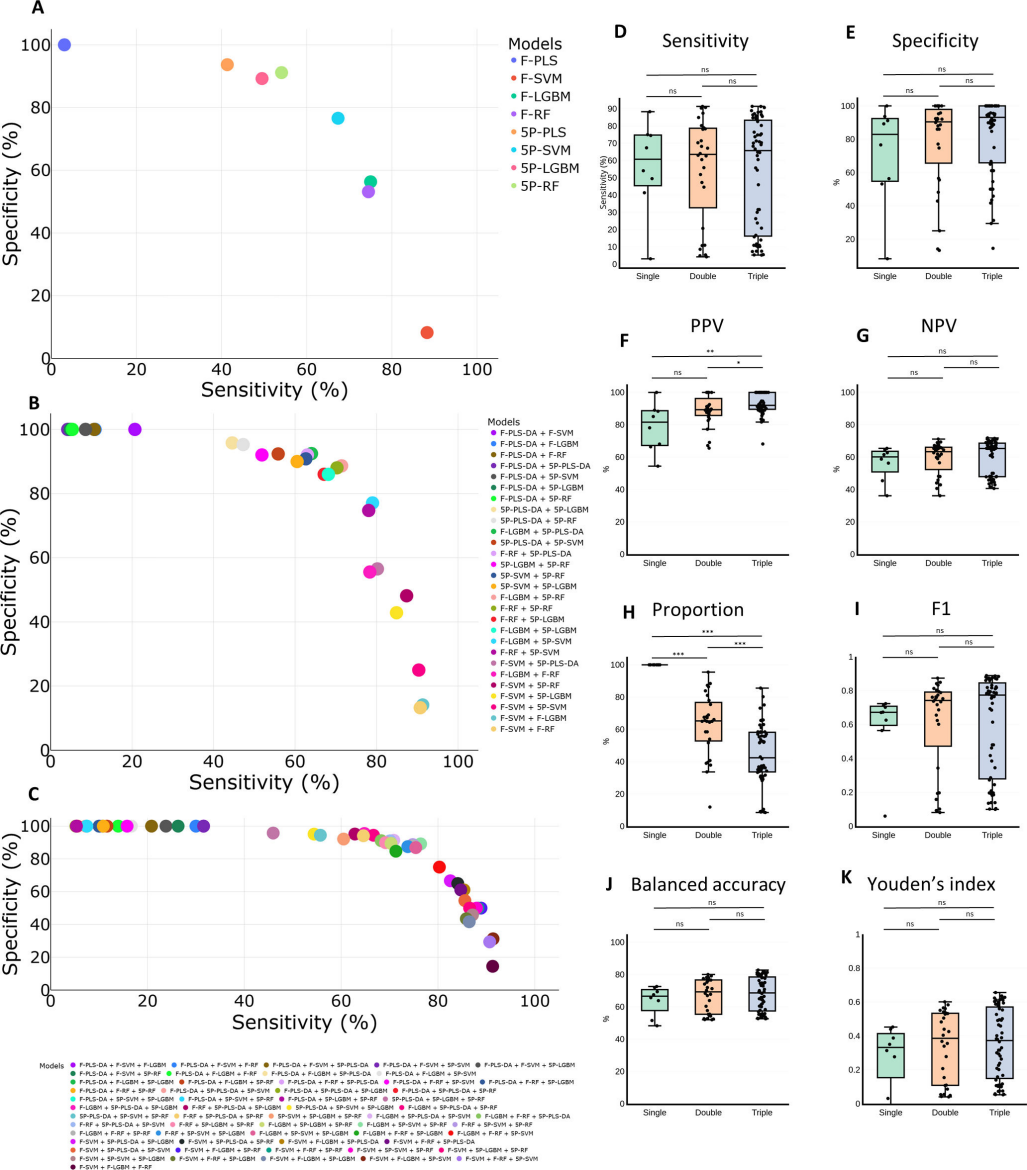
Plot of accuracy values obtained from validation of the first model (Training 1) with Mayo Clinic/ARLG isolates. (**A**) Scatter plot of sensitivity and specificity for the eight single models. (**B**) Scatter plot of sensitivity and specificity for the 28 pairwise combinations. (**C**) Scatter plot of sensitivity and specificity for the 56 triplet combinations. (**D**) Boxplot of sensitivity comparing single and ensemble models. (**E**) Boxplot of specificity comparing single and ensemble models. (**F**) Boxplot of PPV comparing single and ensemble models. (**G**) Boxplot of NPV comparing single and ensemble models. (**H**) Boxplot of the proportion of samples included in each model that yielded the same identification result by all models. (**I**) Boxplot of F1 score comparing single and ensemble models. (**J**) Boxplot of balanced accuracy comparing single and ensemble models. (**K**) Boxplot of Youden’s index comparing single and ensemble models.

The 10-fold cross-validation of the eight single models yielded 84% and 91% correct validation for models based on full spectra (F-PLS-DA, F-SVM, F-LGBM, and F-RF) and between 88% and 91% for models based on the five-peak matrix (5P-PLS-DA, 5P-SVM, 5P-LGBM, and 5P-RF). After performing external validation with the Mayo Clinic and ARLG data sets (*N* = 354), the models with the highest sensitivity and specificity were 5P-PLS-DA (sensitivity = 41% and specificity = 94%) and 5P-RF (sensitivity = 54% and specificity = 91%) (Fig. 3A). By pairwise combination, no model obtained both sensitivity and specificity higher than 80% (Fig. 3B). Considering models with specificity higher than 90% and the highest possible sensitivity, those with the best performance were F-LGBM + 5P-PLS-DA (sensitivity = 64%, specificity = 93%, and F1 = 0.75) and F-RF + 5P-PLS-DA (sensitivity = 63%, specificity = 92%, and F1 = 0.75). As observed before, no model in the triplet combination reached both sensitivity and specificity greater than 80% (Fig. 3C). Considering models with specificity higher than 90% and the highest possible sensitivity, those with the best performance were F-LGBM + 5P-PLS-DA + 5P-LGBM (sensitivity = 65%, specificity = 96%, and F1 = 0.77), F-LGBM + 5P-PLS-DA + 5P-RF (sensitivity = 67%, specificity = 94%, and F1 = 0.78), and F-LGBM + 5P-PLS-DA + 5P-SVM (sensitivity = 71%, specificity = 91%, and F1 = 0.80). Interestingly, the PLS-DA algorithm was present in all ensembles with high specificity. Detailed values for all models are provided in Table S2.

By application of ensemble models, all accuracy values increased (Fig. 3D through K), with PPV being the only one with a statistically significant increase (*P* < 0.05). On the other hand, the proportion of samples that met criteria for evaluation (i.e., same identification by all the models) decreased when more models were included in the ensemble (Fig. 3H).

### Model validation from Training 2

**Fig 4:**
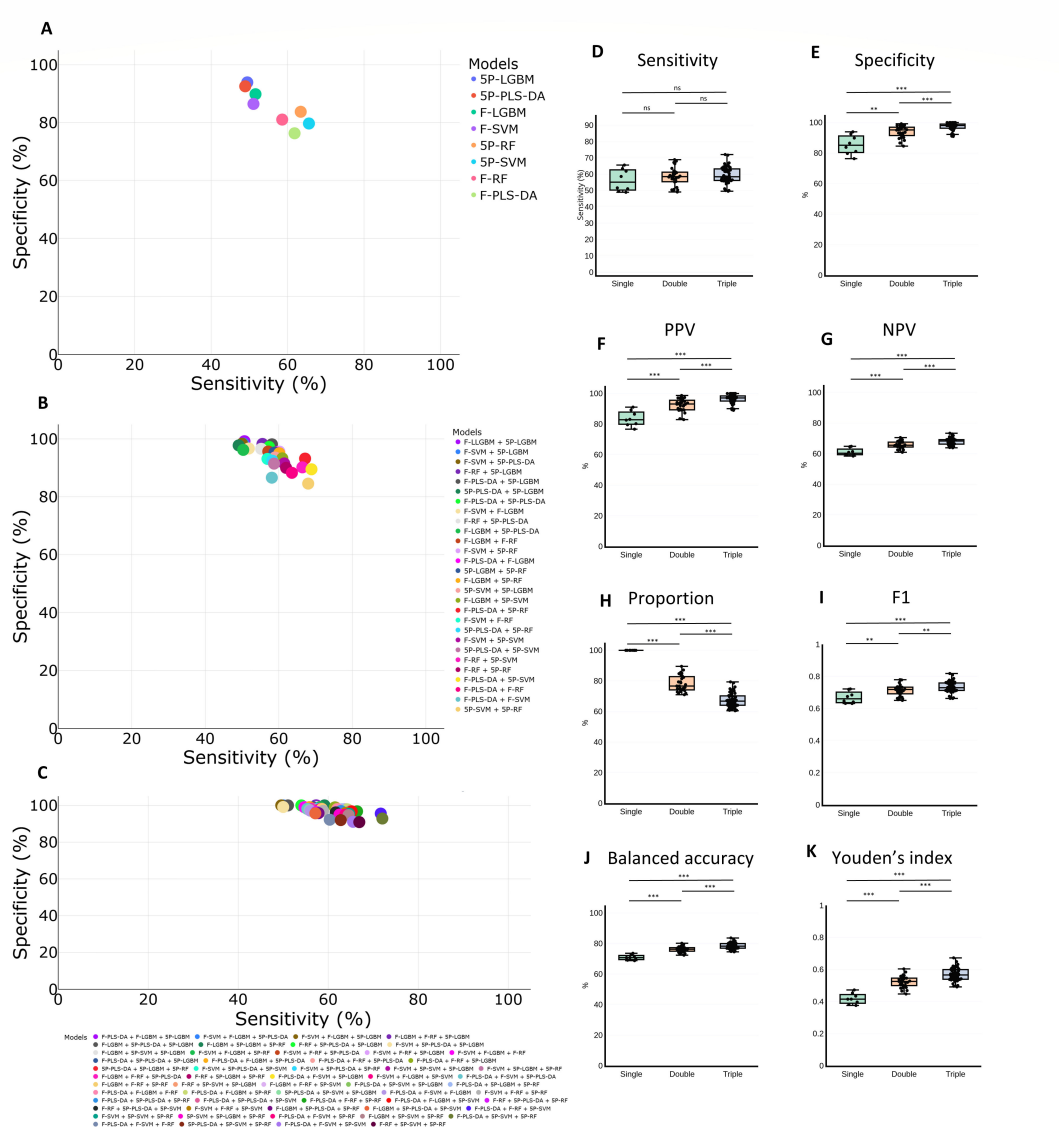
Plot of accuracy values obtained from validation of the second model (Training 2). (**A**) Scatter plot of sensitivity and specificity for the eight single models created. (**B**) Scatter plot of sensitivity and specificity for the 28 pairwise combinations. (**C**) Scatter plot of sensitivity and specificity for the 56 triplet combinations. (**D**) Boxplot of sensitivity comparing single and ensemble models. (**E**) Boxplot of specificity comparing single and ensemble models. (**F**) Boxplot of PPV comparing single and ensemble models. (**G**) Boxplot of NPV comparing single and ensemble models. (**H**) Boxplot of proportion of samples included in each model that yielded the same identification result by all models. (**I**) Boxplot of F1 score comparing single and ensemble models. (**J**) Boxplot of balanced accuracy comparing single and ensemble models. (**K**) Boxplot of Youden’s index comparing single and ensemble models.

In the second training set (Training 2), which included 20 isolates from the Mayo Clinic/ARLG collections, the 10-fold cross-validation was between 75% and 87% for models based on full spectra (F-PLS-DA, F-SVM, F-LGBM, and F-RF) and between 88% and 91% for models based on the five-peak matrix (5P-PLS-DA, 5P-SVM, 5P-LGBM, and 5P-RF). They were validated with the remaining isolates (*N* = 334). Among the individual models, those with the best performance were 5P-RF (sensitivity = 63%, specificity = 84%, and F1 = 0.72) and 5P-SVM (sensitivity = 66%, specificity = 80%, and F1 = 0.72) (Fig. 4A). By pairwise combination, sensitivity (range: 49%–69%) and specificity (range: 85%–99%) were similar across all models (Fig. 4B). According to the F1 index, the best pairwise combinations were F-PLS-DA + 5P-RF (F1 = 0.78), F-SVM + 5P-RF (F1 = 0.73), and F-LGBM + 5P-SVM (F1 = 0.73). With the triplet combination, a similar value was obtained among all models for sensitivity (50%–72%) and specificity (91%–100%) (Fig. 4C), with the outstanding combinations being F-PLS-DA + F-RF + 5P-SVM (sensitivity = 72%, specificity = 96%, and F1 = 0.82) and F-PLS-DA + 5P-SVM + 5P-RF (sensitivity = 72%, specificity = 93%, and F1 = 0.81). Detailed values for all models are provided in Table S3.

By construction of ensemble models with this second group (Training 2), all accuracy values increased (Fig. 4D through K), except sensitivity (Fig. 4D). As observed before, the proportion of samples that met the inclusion criteria (i.e., same result by all algorithms) decreased when more models were included in the ensemble (Fig. 4H).

### Comparison of training groups and MALDI Biotyper KPC module

To evaluate the improvement of accuracy by integration of heterogeneous data into the first training set (Training 1), 20 isolates from Mayo Clinic/ARLG collections were added, creating the Training 2 group. This new training group obtained higher results in all metrics evaluated (Fig. 4D through K). This increase was statistically significant (*P* < 0.05) in specificity between triple combinations (83% vs 97%), in PPV between triple combinations (93% vs 97%), in NPV between double (59% vs 66%) and triple combinations (59% vs 68%), in balanced accuracy between double (67% vs 76%) and triple combinations (68% vs 78%), and in proportion of isolates included between double (63% vs 78%) and triple combinations (45% vs 67%). The MALDI Biotyper KPC detection module obtained a positive result in 63/196 KPC isolates (sensitivity = 32%), with no false-positive results (specificity = 100%). The machine learning classification models increased sensitivity (*P* < 0.05) compared with the KPC module.

### Peak analysis and *in silico* identification

For tentative identification of relevant peaks reported in the literature and those used in this study, several databases were used for matching detected peaks with theoretically predicted protein masses specific for *K. pneumoniae*. Overall, 71 peaks were identified (Table S4). Among them, 31 (44%) corresponded to ribosomal proteins (large subunit, RL and small subunit, RS) and were present in all study isolates. The following ribosomal peaks previously reported as indicative of carbapenem-resistant *K. pneumoniae* (Table 1) (10, 24–26) were present in all resistant and susceptible isolates from this study: 2,182 *m*/*z* (RL36 [M + 2H]^2+^), 4,365 *m*/*z* (RL36 [M + H]^+^), 4,496 *m*/*z* (RL27 [M + H]^+^), 4,768 *m*/*z* (RS17 [M + 2H]^2+^), 5,379 *m*/*z* (RL34 [M + H]^+^), 6,288 *m*/*z* (RL33 [M + CH_3_]^+^), 7,159 *m*/*z* (RL35 [M + H]^+^), 9,540 *m*/*z* (RS17 [M + 2H]^2+^), 10,285 *m*/*z* (RS19 [M + H]^+^), and 10,580 *m*/*z* (RL25 [M + H]^+^).

**TABLE 1:** Peaks reported in literature, presumptive identification of these peaks, and detection of these peaks among study isolates^*a*^^,^^*b*^

Peak (*m/z*)	Protein	Presence in this study	Relation with carbapenem resistance or the presence of *bla*_KPC_ in this study	Previous studies (presence)	Reference
2,180	RL36 [M + 2H]^2+^	100%	No	Carbapenem R	24
2,260	NI	100%	No	Carbapenem R	10
2,480	NI	0%	NA	Carbapenem R	9
2,636	NI	65%	No	Carbapenem R (52%)	24, 25
2,750	NI	16%	No	Carbapenem R	24
2,980	NI	100% in Mayo Clinic/ARLG collection, 0% in HGM collection	No	Carbapenem R	24
3,040	NI	35%	No	Carbapenem R	24
3,057	NI	7%	No	Carbapenem S (55%)	26
3,084	NI	29%	No	Carbapenem S (59%)	26
3,372	NI	32%	KPC	NT	This study
3,514	Glycosidase (Uniprot: A0AB73WH79) [M + H]^+^	0%	NA	Carbapenem R	8
3,651	NI	12%	No	Carbapenem S (30%)	26
3,958	NI	0%	NA	Carbapenem R (45%)	26
4,362	RL36 [M + H]^+^	100%	No	Carbapenem R (45%)	24, 25
4,450	Osmotically inducible protein Y [M + 2H]^2+^	100%	No	Carbapenem R	10
4,480	NI	0%	NA	Carbapenem R	10
4,497	RL27 [M + 2H]^2+^	100%	No	Carbapenem R (96%)	26
4,521	NI	9%	No	KPC-2, ST11. Carbapenem R	24, 27
4,540	NI	100%	No	Carbapenem R	10
4,600	NI	11%	No	Carbapenem R	10
4,768	RS17 [M + 2H]^2+^	100%	No	Carbapenem R (54%)	25
4,920	NI	13%	No	Carbapenem R	10
4,967	NI	100%	No	Carbapenem R	9
5,154	NI	28%	No	Carbapenem S (81%)	26
5,251	NI	37%	KPC	NT	This study
5,280	Stationary phase-induced ribosome-associated protein [M + H]^+^	100%	No	Carbapenem R	24
5,379	RL34 [M + 2H]^2+^	100%	No	Carbapenem R (54%)	25
5,820	NI	67%	No	Carbapenem R	24
5,884	NI	100%	No	Carbapenem R (77%) KPC	10, 26, 28
5,939	Heat shock protein HspQ [M + 2H]^2+^	100%	No	Carbapenem R	10
6,017	NI	42%	KPC	NT	This study
6,116	NI	0%	NA	Carbapenem S (51%)	26
6,150	Uncharacterized protein YmdF [M + H]^+^	100%	No	Carbapenem R	24
6,288	RL33 [M + CH_3_]^+^	100%	No	Carbapenem R (76%)	25
6,515	NI	30%	No	Carbapenem R (56%)	26
6,684	NI	0%	NA	KPC	28
7,158	RL35 [M + H]^+^	100%	No	Carbapenem R (89%)	25
7,578	NI	0%	NA	KPC	28
7,705	NI	100%	No	Carbapenem R (80%, 95%)	25, 26
8,054	NI	0%	NA	KPC	28
8,744	NI	0%	NA	KPC	28
9,478	DNA-binding protein HU-alpha [M + H]^+^	100%	No	Carbapenem R (82%)	25
9,541	RS17 [M + H]^+^	100%	No	Carbapenem R (76%)	25
9,840	NI	13%	No	Carbapenem R	10
9,852	Ethanolamine utilization protein EutN [M + H]^+^	84%	No	Carbapenem R (85%)	26
10,287	RS19 [M + H]^+^	100%	No	Carbapenem R (76%)	25
10,580	RL25 [M + H]^+^	100%	No	Carbapenem R	10
10,883	Uncharacterized protein YqjD [M + H]^+^	100%	No	KPC	28
11,767	NI	0%	NA	KPC	28
12,260	NI	0%	NA	KPC	28
12,362	NI	0%	NA	Carbapenem R	9
12,506	NI	0%	NA	Carbapenem R	9
12,855	NI	0%	NA	Carbapenem R	9
13,004	NI	0%	NA	KPC	28
13,366	NI	0%	NA	KPC	28
14,445	NI	0%	NA	KPC	28
14,790	NI	0%	NA	Carbapenem R	9
15,139	NI	0%	NA	KPC	28
15,730	NI	0%	NA	Carbapenem R	9
16,109	NI	0%	NA	KPC	28
16,176	NI	0%	NA	Carbapenem R	9
16,218	NI	0%	NA	Carbapenem R	9
16,758	NI	0%	NA	Carbapenem R	9
16,919	NI	0%	NA	Carbapenem R	9
17,091	NI	0%	NA	Carbapenem R	9
17,489	NI	0%	NA	KPC	28
18,142	NI	0%	NA	Carbapenem R	9
18,582	NI	0%	NA	KPC	28
18,998	NI	0%	NA	Carbapenem R	9
19,095	NI	0%	NA	Carbapenem R	9

^*a*^ The percentage of previously described peaks in the study isolates is indicated. Due to minor technical variability, peak shifts of ±5 *m*/*z* were considered the same peak.

^*b*^ NA: not applicable; NT, not tested; NI, not identified; R, resistant; RL, ribosomal protein large subunit; RS, ribosomal protein small subunit; S, susceptible; ST, sequence type.

The remaining 40 peaks (56%) corresponded to other proteins of *K. pneumoniae* (Table S4). Among them, peaks reported in previous studies as present in carbapenem-resistant isolates were found in both resistant and susceptible isolates in this study (Table 1), including 4,450 *m*/*z* (osmotically inducible protein Y [M + 2H]^2+^), 5,280 *m*/*z* (stationary phase-induced ribosome-associated protein [M + H]^+^), 5,940 *m*/*z* (heat shock protein HspQ [M + 2H]^2+^), 6,152 *m*/*z* (uncharacterized protein YmdF [M + H]^+^), 9,478 *m*/*z* (DNA-binding protein HU-alpha [M + H]^+^), 9,849 *m*/*z* (ethanolamine utilization protein EutN [M + H]^+^), and 10,885 *m*/*z* (uncharacterized protein YqjD [M + H]^+^). In addition, peaks corresponding to the known pKpQIL_p019 protein (5,555 and 11,109 *m*/*z*) were detected in 95/237 (40%) KPC-positive isolates.

Among the five peaks used in the models here, aside from the known 5,555 and 11,109 *m*/*z* peaks, the others (3,372 *m*/*z*, 5,251 *m*/*z*, and 6,017 *m*/*z*) were not identified by the previous annotation method and have not been reported. With all the information regarding peak identification, an interactive annotated spectrum for *K. pneumoniae* was constructed (File S1).

With regard to the peaks observed in previous studies that were not identified, 31 were not detected in this collection, 9 were found in ≤30% isolates, and the others were either detected in all isolates or indifferently distributed among KPC-positive and KPC-negative isolates (Table 1).

### Inferring the reason for low sensitivity

To attempt to understand the reason behind the low sensitivity, KPC isolates (*n* = 20) with WGS information that yielded false negative results by the most accurate predictive models were selected for (i) genome exploration (sequence types, resistance genes, virulence genes, capsular type, and serotype) to find potentially associated characteristics and (ii) re-analysis by MALDI-TOF MS (following the same experimental conditions used for the initial analysis) and predictive models. No common trends were found regarding sequence types, resistance genes, virulence genes, or capsular type. With repeat analysis by MALDI-TOF MS, however, five (25%) isolates were correctly identified as KPC-producing isolates using ensemble triple models, and another five (25%) were correctly classified by at least one model.

## DISCUSSION

The first single models developed in this study and validated using an external data set from another location yielded variable results in terms of sensitivity and specificity. Although using some models, specificity reached >90%, sensitivity for KPC-producing isolates was low (54%). The strategy of building ensemble models by the combination of two and three algorithms resulted in an increase in sensitivity and specificity, although no statistically significant differences were obtained, except for an increase in PPV. This could be explained by different ML algorithms recognizing different patterns among data, with the combination resulting in a comprehensive interpretation of spectral features (29). Even if sensitivity remained low, with values ~70% for some combinations, application of ML models significantly improved sensitivity in comparison with the MALDI Biotyper KPC module, which is based on the single peak of 11,109 *m*/*z*, demonstrating that the use of more data from mass spectra leads to the detection of a higher number of KPC-producing isolates. On the other hand, as more algorithms were included in the ensemble, a lower proportion of isolates was classifiable, since the criterion of only accepting isolates with the same results in all models of the ensemble was applied (and only met in 60%–75% of isolates in the best models).

Following this observation, and considering that the first models tested were trained with isolates from HGM, a new training set including representatives from the Mayo Clinic/ARLG collections was used. This was important to increase heterogeneity of the data to obtain models that generalize better with external data sets, a strategy successfully used in other studies (30). The addition of only 20 isolates from the Mayo Clinic and ARLG collections to the initial training set resulted in an increase in almost all metrics evaluated for pairwise and triple ensemble models. However, sensitivity remained low (maximum, 72%). In addition, the proportion of samples considered for result analysis also increased. In this context, ensemble models could be particularly useful in situations that aim to avoid false-positive results for KPC detection, even if there is a loss of sensitivity as a result of gain in specificity.

To further explore peaks reported in the literature and those identified here, *in silico* prediction of peak characterization was performed using different databases to calculate the theoretical mass of proteins specific for *K. pneumoniae* (also considering known PTMs), as other studies have done with other species (31, 32). Peaks could correspond to conserved proteins present in most *K. pneumoniae* isolates and especially ribosomal proteins, which are used by MALDI-TOF MS for species identification (33). However, some peaks have been reported as biomarkers for carbapenem-resistant isolates (Table 1), with no identification provided (24–26). The fact that these biomarker peaks were found in all isolates from the current collection, which includes isolates from Spain, the United States, Latin America (Colombia and Chile) and Lebanon (12), reinforces their nature as conserved proteins. The difference in this observation for all peaks (identified and not identified) with previous studies could be explained by most studies being from Asian countries, with geographical variation compared to the isolates studied here, and/or the spectral processing steps in other studies could vary, making selection of peaks not comparable.

With putative peaks identified, an annotated mass spectrum specific for *K. pneumoniae* was constructed, representing, to our knowledge, the first public annotation made on a MALDI-TOF MS spectrum (File S1). Despite being constructed based on a collection of isolates from different regions, it should be noted that other *K. pneumoniae* isolates may show minor shifts and variations as a result of natural variability. This theoretical annotation could guide future studies focusing on mass spectra of *K. pneumoniae*, by adding a biological perspective to the peaks detected by MALDI-TOF MS. Interestingly, three unidentified peaks (3,372, 5,251, and 6,017 *m*/*z*) were shown to be mostly present in KPC-producing isolates; these need to be confirmed with other collections, since they may reflect clonal or geographic signatures rather than resistance determinants, which may account for differences in their presence across studies.

With the aim of further exploring the reasons behind low sensitivity and to assess if the cause of misclassification could be a biological or technical issue, a subset of 20 KPC isolates that had yielded false negative results was selected for genome exploration and repeat MALDI-TOF MS analysis. First, by genome characteristics analyzed, no common traits were found, at least those related to resistance and virulence genes or sequence and capsular types. However, half (10/20) yielded a positive result when re-analyzed and run through the models again. This suggests that technical variability during MALDI-TOF MS spectral acquisition may lead to low resolution of important peaks and be interpreted as false-negative results by predictive models. While calibration, spectral acquisition parameters, and sample preparation were performed under the same conditions, variability may exist between runs and/or MALDI-TOF MS instruments, which may influence signal detection. It cannot be excluded that, in some cases, the absence of peaks reflects intrinsic biological rather than technical variability. In the clinical setting, repeat testing could be accomplished using multiple spots on the MALDI-TOF MS target for initial identification or repeat testing on different runs, but this would increase workload, cost, and for the latter, turnaround time, considering that *K. pneumoni*ae is a commonly isolated species. Future approaches to reduce technical variability could include the use of automated MALDI-TOF-spotting systems.

There are several limitations of this study. The study used different isolate collections that had different susceptibility profiles. The ARLG collection included all carbapenem-resistant isolates (KPC positive and negative), whereas the Mayo Clinic collection consisted entirely of carbapenem-susceptible isolates. In addition, KPC presence was defined using different approaches: gene presence by whole-genome analysis for ARLG isolates and phenotypic detection for HGM isolates. This may introduce subtle differences in resistance classification. Spectral acquisition was performed by on-plate protein extraction of single subcultures from frozen stocks, which may have influenced results compared to performing two subcultures. Future studies could evaluate optimization for higher resolution using protein extraction or subcultures passaged twice. Another limitation of this study is that repeat testing was not performed on all study isolates.

Several practical aspects must be considered for the described technique to be made suitable for clinical laboratories. Spectral acquisition procedures may need to be defined to reduce technical variability, and additional spectral quality control, beyond that used for identification, may need to be implemented. In addition, integration of classification models with MALDI-TOF MS spectra acquisition software would require the development of an informatics workflow to automate the process from spectral acquisition to result. In conclusion, this study showed that the construction of ensemble models with diverse strain collections leads to an increase in accuracy for MALDI-TOF MS-based detection of KPC-positive *K. pneumoniae*, representing a step toward the detection of resistance mechanisms by proteomic analysis. While sensitivity remains a challenge, possibly affected by technical variability during mass spectral acquisition, it was improved in comparison with current MALDI-TOF-based methods. Characterization of protein peaks could inform future studies, determining which signals represent specific peaks in need of further investigation as potential diagnostic biomarkers.

## Data Availability

WGS data from ARLG isolates were previously reported (18, 19) and can be found in GenBank (accession number PRJNA658369). Raw mass spectra profiles can be requested from the corresponding author upon reasonable request.

## SUPPLEMENTAL MATERIAL

File S1 jcm.01466-25-s0001.html Annotated mass spectrum of *Klebsiella pneumoniae*, showing theoretical identified proteins with single and double charges.

Table S1 jcm.01466-25-s0002.docx Isolates included in the study were obtained from three different collections: HGM, Hospital Gregorio Marañón; ARLG, Antibacterial Resistance Leadership Group; and Mayo Clinic.

Table S2 jcm.01466-25-s0003.docx Detailed values for validation of Training 1 group for each individual predictive model and ensemble by pairwise and triple combinations. Data for each ensemble type are sorted by decreasing specificity.

Table S3 jcm.01466-25-s0004.docx Detailed values for validation of Training 2 group for each individual predictive model and ensemble by pairwise and triple combinations. Data for each ensemble type are sorted by decreasing specificity.

Table S4 jcm.01466-25-s0005.docx List of the 71 peaks for which a presumptive identification was obtained, including the name of the protein and accession number to Uniprot database.

ASM does not own the copyrights to Supplemental Material that may be linked to, or accessed through, an article. The authors have granted ASM a non-exclusive, world-wide license to publish the Supplemental Material files. Please contact the corresponding author directly for reuse.
