# Supplementary material for: Prediction of KPC-producing Klebsiella pneumoniae by MALDI-TOF MS, ensemble learning, and spectral peak annotation
Source: J Clin Microbiol. 2026 Mar 30;64(5):e01466-25. doi: 10.1128/jcm.01466-25 (PMC13170361; doi:10.1128/jcm.01466-25)
Supplement: Table S1 — Isolates included in the study were obtained from three different collections: HGM, Hospital Gregorio Marañón; ARLG, Antibacterial Resistance Leadership Group; and Mayo Clinic. [file jcm.01466-25-s0002.docx]

**Table S1.** Isolates included in the study obtained from three different collections. HGM, Hospital Gregorio Marañón; ARLG, Antibacterial Resistance Leadership Group; N/A, Not Applicable; NT, Not Tested.

| **Isolate ID** | **Collection** | **KPC presence** | **KPC type** |
| --- | --- | --- | --- |
| 82 | HGM | No | N/A |
| 127 | HGM | No | N/A |
| 133 | HGM | No | N/A |
| 589 | HGM | No | N/A |
| 643 | HGM | No | N/A |
| 879 | HGM | No | N/A |
| 926 | HGM | No | N/A |
| 1019 | HGM | No | N/A |
| 1068 | HGM | No | N/A |
| 1082 | HGM | No | N/A |
| 1115 | HGM | No | N/A |
| 1139 | HGM | No | N/A |
| 1150 | HGM | No | N/A |
| 1320 | HGM | No | N/A |
| 1334 | HGM | No | N/A |
| 1593 | HGM | No | N/A |
| 1688 | HGM | No | N/A |
| 1970 | HGM | No | N/A |
| 2088 | HGM | No | N/A |
| 2116 | HGM | No | N/A |
| 2416 | HGM | No | N/A |
| 2461 | HGM | No | N/A |
| 2523 | HGM | No | N/A |
| 2786 | HGM | No | N/A |
| 2799 | HGM | No | N/A |
| 2893 | HGM | No | N/A |
| 2905 | HGM | No | N/A |
| 3078 | HGM | No | N/A |
| 3122 | HGM | No | N/A |
| 3238 | HGM | No | N/A |
| 3327 | HGM | No | N/A |
| 3347 | HGM | No | N/A |
| 3452 | HGM | No | N/A |
| 3459 | HGM | No | N/A |
| 3493 | HGM | No | N/A |
| 3515 | HGM | No | N/A |
| 3839 | HGM | No | N/A |
| 3952 | HGM | No | N/A |
| 3978 | HGM | No | N/A |
| 4025 | HGM | No | N/A |
| 95 | HGM | Yes | NT |
| 281 | HGM | Yes | NT |
| 312 | HGM | Yes | NT |
| 366 | HGM | Yes | NT |
| 411 | HGM | Yes | NT |
| 422 | HGM | Yes | NT |
| 460 | HGM | Yes | NT |
| 469 | HGM | Yes | NT |
| 489 | HGM | Yes | NT |
| 554 | HGM | Yes | NT |
| 564 | HGM | Yes | NT |
| 567 | HGM | Yes | NT |
| 617 | HGM | Yes | NT |
| 623 | HGM | Yes | NT |
| 772 | HGM | Yes | NT |
| 821 | HGM | Yes | NT |
| 1016 | HGM | Yes | NT |
| 1361 | HGM | Yes | NT |
| 1380 | HGM | Yes | NT |
| 1381 | HGM | Yes | NT |
| 1392 | HGM | Yes | NT |
| 1472 | HGM | Yes | NT |
| 1550 | HGM | Yes | NT |
| 1600 | HGM | Yes | NT |
| 1603 | HGM | Yes | NT |
| 1646 | HGM | Yes | NT |
| 1883 | HGM | Yes | NT |
| 2063 | HGM | Yes | NT |
| 2196 | HGM | Yes | NT |
| 2497 | HGM | Yes | NT |
| 2565 | HGM | Yes | NT |
| 2643 | HGM | Yes | NT |
| 2686 | HGM | Yes | NT |
| 2978 | HGM | Yes | NT |
| 3080 | HGM | Yes | NT |
| 3189 | HGM | Yes | NT |
| 3202 | HGM | Yes | NT |
| 3236 | HGM | Yes | NT |
| 3387 | HGM | Yes | NT |
| 3768 | HGM | Yes | NT |
| 4352 | HGM | Yes | NT |
| Kp-001 | Mayo Clinic | No | N/A |
| Kp-002 | Mayo Clinic | No | N/A |
| Kp-003 | Mayo Clinic | No | N/A |
| Kp-004 | Mayo Clinic | No | N/A |
| Kp-005 | Mayo Clinic | No | N/A |
| Kp-006 | Mayo Clinic | No | N/A |
| Kp-007 | Mayo Clinic | No | N/A |
| Kp-008 | Mayo Clinic | No | N/A |
| Kp-010 | Mayo Clinic | No | N/A |
| Kp-012 | Mayo Clinic | No | N/A |
| Kp-013 | Mayo Clinic | No | N/A |
| Kp-014 | Mayo Clinic | No | N/A |
| Kp-015 | Mayo Clinic | No | N/A |
| Kp-016 | Mayo Clinic | No | N/A |
| Kp-017 | Mayo Clinic | No | N/A |
| Kp-018 | Mayo Clinic | No | N/A |
| Kp-019 | Mayo Clinic | No | N/A |
| Kp-020 | Mayo Clinic | No | N/A |
| Kp-021 | Mayo Clinic | No | N/A |
| Kp-022 | Mayo Clinic | No | N/A |
| Kp-025 | Mayo Clinic | No | N/A |
| Kp-026 | Mayo Clinic | No | N/A |
| Kp-028 | Mayo Clinic | No | N/A |
| Kp-029 | Mayo Clinic | No | N/A |
| Kp-030 | Mayo Clinic | No | N/A |
| Kp-031 | Mayo Clinic | No | N/A |
| Kp-032 | Mayo Clinic | No | N/A |
| Kp-033 | Mayo Clinic | No | N/A |
| Kp-034 | Mayo Clinic | No | N/A |
| Kp-035 | Mayo Clinic | No | N/A |
| Kp-037 | Mayo Clinic | No | N/A |
| Kp-039 | Mayo Clinic | No | N/A |
| Kp-040 | Mayo Clinic | No | N/A |
| Kp-041 | Mayo Clinic | No | N/A |
| Kp-043 | Mayo Clinic | No | N/A |
| Kp-044 | Mayo Clinic | No | N/A |
| Kp-045 | Mayo Clinic | No | N/A |
| Kp-046 | Mayo Clinic | No | N/A |
| Kp-047 | Mayo Clinic | No | N/A |
| Kp-048 | Mayo Clinic | No | N/A |
| Kp-049 | Mayo Clinic | No | N/A |
| Kp-052 | Mayo Clinic | No | N/A |
| Kp-055 | Mayo Clinic | No | N/A |
| Kp-056 | Mayo Clinic | No | N/A |
| Kp-057 | Mayo Clinic | No | N/A |
| Kp-058 | Mayo Clinic | No | N/A |
| Kp-059 | Mayo Clinic | No | N/A |
| Kp-060 | Mayo Clinic | No | N/A |
| Kp-061 | Mayo Clinic | No | N/A |
| Kp-063 | Mayo Clinic | No | N/A |
| Kp-064 | Mayo Clinic | No | N/A |
| Kp-065 | Mayo Clinic | No | N/A |
| Kp-066 | Mayo Clinic | No | N/A |
| Kp-067 | Mayo Clinic | No | N/A |
| Kp-068 | Mayo Clinic | No | N/A |
| Kp-069 | Mayo Clinic | No | N/A |
| Kp-070 | Mayo Clinic | No | N/A |
| Kp-071 | Mayo Clinic | No | N/A |
| Kp-072 | Mayo Clinic | No | N/A |
| Kp-073 | Mayo Clinic | No | N/A |
| Kp-074 | Mayo Clinic | No | N/A |
| Kp-075 | Mayo Clinic | No | N/A |
| Kp-076 | Mayo Clinic | No | N/A |
| Kp-078 | Mayo Clinic | No | N/A |
| Kp-079 | Mayo Clinic | No | N/A |
| Kp-080 | Mayo Clinic | No | N/A |
| Kp-081 | Mayo Clinic | No | N/A |
| Kp-082 | Mayo Clinic | No | N/A |
| Kp-083 | Mayo Clinic | No | N/A |
| Kp-084 | Mayo Clinic | No | N/A |
| Kp-085 | Mayo Clinic | No | N/A |
| Kp-086 | Mayo Clinic | No | N/A |
| Kp-088 | Mayo Clinic | No | N/A |
| Kp-089 | Mayo Clinic | No | N/A |
| Kp-090 | Mayo Clinic | No | N/A |
| Kp-091 | Mayo Clinic | No | N/A |
| Kp-092 | Mayo Clinic | No | N/A |
| Kp-096 | Mayo Clinic | No | N/A |
| Kp-097 | Mayo Clinic | No | N/A |
| Kp-098 | Mayo Clinic | No | N/A |
| Kp-099 | Mayo Clinic | No | N/A |
| Kp-100 | Mayo Clinic | No | N/A |
| Kp-101 | Mayo Clinic | No | N/A |
| Kp-102 | Mayo Clinic | No | N/A |
| ARLG-7444 | ARLG | No | N/A |
| ARLG-7309 | ARLG | No | N/A |
| ARLG-7314 | ARLG | No | N/A |
| ARLG-7316 | ARLG | No | N/A |
| ARLG-7317 | ARLG | No | N/A |
| ARLG-7318 | ARLG | No | N/A |
| ARLG-7319 | ARLG | No | N/A |
| ARLG-7321 | ARLG | No | N/A |
| ARLG-7322 | ARLG | No | N/A |
| ARLG-7323 | ARLG | No | N/A |
| ARLG-7325 | ARLG | No | N/A |
| ARLG-7326 | ARLG | No | N/A |
| ARLG-7327 | ARLG | No | N/A |
| ARLG-7331 | ARLG | No | N/A |
| ARLG-7332 | ARLG | No | N/A |
| ARLG-7333 | ARLG | No | N/A |
| ARLG-7334 | ARLG | No | N/A |
| ARLG-7335 | ARLG | No | N/A |
| ARLG-7336 | ARLG | No | N/A |
| ARLG-7337 | ARLG | No | N/A |
| ARLG-7338 | ARLG | No | N/A |
| ARLG-7339 | ARLG | No | N/A |
| ARLG-7340 | ARLG | No | N/A |
| ARLG-7341 | ARLG | No | N/A |
| ARLG-7343 | ARLG | No | N/A |
| ARLG-7344 | ARLG | No | N/A |
| ARLG-7345 | ARLG | No | N/A |
| ARLG-7346 | ARLG | No | N/A |
| ARLG-7348 | ARLG | No | N/A |
| ARLG-7350 | ARLG | No | N/A |
| ARLG-7228 | ARLG | Yes | KPC2 |
| ARLG-7683 | ARLG | No | N/A |
| ARLG-7568 | ARLG | No | N/A |
| ARLG-7794 | ARLG | Yes | KPC2 |
| ARLG-8120 | ARLG | No | N/A |
| ARLG-7546 | ARLG | No | N/A |
| ARLG-7724 | ARLG | Yes | KPC2 |
| ARLG-8134 | ARLG | Yes | KPC2 |
| ARLG-7507 | ARLG | Yes | KPC3 |
| ARLG-8064 | ARLG | No | N/A |
| ARLG-8068 | ARLG | No | N/A |
| ARLG-7671 | ARLG | Yes | KPC28 |
| ARLG-7807 | ARLG | Yes | KPC34 |
| ARLG-7818 | ARLG | No | N/A |
| ARLG-7888 | ARLG | No | N/A |
| ARLG-8022 | ARLG | No | N/A |
| ARLG-7645 | ARLG | No | N/A |
| ARLG-6473 | ARLG | No | N/A |
| ARLG-6547 | ARLG | No | N/A |
| ARLG-8087-P | ARLG | Yes | KPC2 |
| ARLG-8091-P | ARLG | Yes | KPC3 |
| ARLG-8099-P | ARLG | Yes | KPC3 |
| ARLG-8101-P | ARLG | Yes | KPC3 |
| ARLG-8107-P | ARLG | Yes | KPC3 |
| ARLG-7711-P | ARLG | Yes | KPC3 |
| ARLG-7713-P | ARLG | Yes | KPC3 |
| ARLG-7712-P | ARLG | Yes | KPC3 |
| ARLG-7509-P | ARLG | Yes | KPC3 |
| ARLG-7510-P | ARLG | Yes | KPC2 |
| ARLG-7513-P | ARLG | Yes | KPC3 |
| ARLG-7517-P | ARLG | Yes | KPC2 |
| ARLG-7520-P | ARLG | Yes | KPC2 |
| ARLG-7521-P | ARLG | Yes | KPC3 |
| ARLG-7522-P | ARLG | Yes | KPC2 |
| ARLG-7525-P | ARLG | Yes | KPC2 |
| ARLG-7527-P | ARLG | Yes | KPC2 |
| ARLG-7528-P | ARLG | Yes | KPC2 |
| ARLG-7532-P | ARLG | Yes | KPC3 |
| ARLG-7829-P | ARLG | Yes | KPC2 |
| ARLG-7832-P | ARLG | Yes | KPC3 |
| ARLG-7839-P | ARLG | Yes | KPC2 |
| ARLG-8109-P | ARLG | Yes | KPC3 |
| ARLG-8111-P | ARLG | Yes | KPC3 |
| ARLG-8127-P | ARLG | Yes | KPC3 |
| ARLG-7714-P | ARLG | Yes | KPC3 |
| ARLG-7716-P | ARLG | Yes | KPC3 |
| ARLG-7717-P | ARLG | Yes | KPC3 |
| ARLG-7718-P | ARLG | Yes | KPC3 |
| ARLG-7535-P | ARLG | Yes | KPC2 |
| ARLG-7536-P | ARLG | Yes | KPC2 |
| ARLG-7537-P | ARLG | Yes | KPC3 |
| ARLG-7544-P | ARLG | Yes | KPC2 |
| ARLG-7547-P | ARLG | Yes | KPC3 |
| ARLG-7548-P | ARLG | Yes | KPC2 |
| ARLG-7550-P | ARLG | Yes | KPC2 |
| ARLG-7551-P | ARLG | Yes | KPC2 |
| ARLG-7552-P | ARLG | Yes | KPC2 |
| ARLG-7553-P | ARLG | Yes | KPC3 |
| ARLG-7529-P | ARLG | Yes | KPC2 |
| ARLG-7847-P | ARLG | Yes | KPC3 |
| ARLG-7849-P | ARLG | Yes | KPC3 |
| ARLG-7799-P | ARLG | Yes | KPC3 |
| ARLG-7801-P | ARLG | Yes | KPC2 |
| ARLG-8135-P | ARLG | Yes | KPC3 |
| ARLG-7541-P | ARLG | Yes | KPC2 |
| ARLG-7837-P | ARLG | Yes | KPC2 |
| ARLG-7851-P | ARLG | Yes | KPC3 |
| ARLG-7675-P | ARLG | Yes | KPC3 |
| ARLG-7576-P | ARLG | Yes | KPC2 |
| ARLG-7556-P | ARLG | Yes | KPC2 |
| ARLG-7582-P | ARLG | Yes | KPC2 |
| ARLG-7584-P | ARLG | No | N/A |
| ARLG-7988-P | ARLG | Yes | KPC3 |
| ARLG-7591-P | ARLG | Yes | KPC2 |
| ARLG-7924-P | ARLG | Yes | KPC3 |
| ARLG-7557-P | ARLG | Yes | KPC3 |
| ARLG-7596-P | ARLG | Yes | KPC2 |
| ARLG-7560-P | ARLG | Yes | KPC2 |
| ARLG-7889-P | ARLG | Yes | KPC3 |
| ARLG-8025-P | ARLG | Yes | KPC3 |
| ARLG-7565-P | ARLG | Yes | KPC2 |
| ARLG-8000-P | ARLG | Yes | KPC2 |
| ARLG-7802-P | ARLG | Yes | KPC3 |
| ARLG-7616-P | ARLG | Yes | KPC3 |
| ARLG-8003-P | ARLG | Yes | KPC2 |
| ARLG-8004-P | ARLG | Yes | KPC3 |
| ARLG-7617-P | ARLG | Yes | KPC2 |
| ARLG-7963-P | ARLG | No | N/A |
| ARLG-7620-P | ARLG | Yes | KPC2 |
| ARLG-7621-P | ARLG | Yes | KPC3 |
| ARLG-7901-P | ARLG | Yes | KPC2 |
| ARLG-7903-P | ARLG | Yes | KPC3 |
| ARLG-7622-P | ARLG | Yes | KPC2 |
| ARLG-7624-P | ARLG | Yes | KPC2 |
| ARLG-7688-P | ARLG | Yes | KPC2 |
| ARLG-7906-P | ARLG | Yes | KPC2 |
| ARLG-8007-P | ARLG | Yes | KPC3 |
| ARLG-8010-P | ARLG | Yes | KPC31 |
| ARLG-7775-P | ARLG | Yes | KPC3 |
| ARLG-7696-P | ARLG | Yes | KPC2 |
| ARLG-7697-P | ARLG | Yes | KPC3 |
| ARLG-7631-P | ARLG | Yes | KPC2 |
| ARLG-7791-P | ARLG | Yes | KPC2 |
| ARLG-7779-P | ARLG | Yes | KPC2 |
| ARLG-7642-P | ARLG | Yes | KPC2 |
| ARLG-7572-P | ARLG | Yes | KPC3 |
| ARLG-7646-P | ARLG | Yes | KPC2 |
| ARLG-7647-P | ARLG | Yes | KPC3 |
| ARLG-7654-P | ARLG | Yes | KPC3 |
| ARLG-7981-P | ARLG | No | N/A |
| ARLG-7982-P | ARLG | Yes | KPC3 |
| ARLG-7656-P | ARLG | Yes | KPC2 |
| ARLG-8020-P | ARLG | Yes | KPC2 |
| ARLG-8021-P | ARLG | Yes | KPC3 |
| ARLG-7983-P | ARLG | Yes | KPC3 |
| ARLG-7673-P | ARLG | Yes | KPC3 |
| ARLG-7729-P | ARLG | Yes | KPC2 |
| ARLG-7735-P | ARLG | Yes | KPC3 |
| ARLG-7916-P | ARLG | Yes | KPC3 |
| ARLG-7996-U | ARLG | Yes | KPC2 |
| ARLG-7618-U | ARLG | Yes | KPC2 |
| ARLG-3495 | ARLG | Yes | KPC2 |
| ARLG-3496 | ARLG | Yes | KPC3 |
| ARLG-3499 | ARLG | Yes | KPC3 |
| ARLG-3503 | ARLG | Yes | KPC3 |
| ARLG-3504 | ARLG | Yes | KPC2 |
| ARLG-3508 | ARLG | Yes | KPC2 |
| ARLG-3510 | ARLG | No | N/A |
| ARLG-3511 | ARLG | Yes | KPC3 |
| ARLG-3517 | ARLG | Yes | KPC2 |
| ARLG-3518 | ARLG | Yes | KPC2 |
| ARLG-3520 | ARLG | Yes | KPC2 |
| ARLG-3523 | ARLG | Yes | KPC3 |
| ARLG-3524 | ARLG | No | N/A |
| ARLG-3527 | ARLG | Yes | KPC3 |
| ARLG-3529 | ARLG | Yes | KPC2 |
| ARLG-3530 | ARLG | Yes | KPC3 |
| ARLG-3532 | ARLG | Yes | KPC3 |
| ARLG-3533 | ARLG | No | N/A |
| ARLG-3534 | ARLG | Yes | KPC3 |
| ARLG-3535 | ARLG | Yes | KPC3 |
| ARLG-3536 | ARLG | No | N/A |
| ARLG-3546 | ARLG | Yes | KPC3 |
| ARLG-3548 | ARLG | Yes | KPC3 |
| ARLG-3553-P | ARLG | Yes | KPC2 |
| ARLG-3567-P | ARLG | Yes | KPC2 |
| ARLG-3575-P | ARLG | Yes | KPC2 |
| ARLG-3576-P | ARLG | Yes | KPC2 |
| ARLG-3578-P | ARLG | Yes | KPC3 |
| ARLG-3591-P | ARLG | Yes | KPC3 |
| ARLG-3594-P | ARLG | Yes | KPC2 |
| ARLG-3595-P | ARLG | No | N/A |
| ARLG-3596-P | ARLG | Yes | KPC2 |
| ARLG-3598-P | ARLG | Yes | KPC3 |
| ARLG-3602-P | ARLG | Yes | KPC2 |
| ARLG-3603-P | ARLG | Yes | KPC3 |
| ARLG-3609-P | ARLG | No | N/A |
| ARLG-3611-P | ARLG | Yes | KPC3 |
| ARLG-3612-P | ARLG | Yes | KPC2 |
| ARLG-3613-P | ARLG | Yes | KPC3 |
| ARLG-3616-P | ARLG | Yes | KPC2 |
| ARLG-3617-P | ARLG | Yes | KPC2 |
| ARLG-3618-P | ARLG | Yes | KPC3 |
| ARLG-3621-P | ARLG | Yes | KPC2 |
| ARLG-3622-P | ARLG | Yes | KPC3 |
| ARLG-3623-P | ARLG | Yes | KPC2 |
| ARLG-3626-P | ARLG | Yes | KPC2 |
| ARLG-3627-P | ARLG | No | N/A |
| ARLG-3628-P | ARLG | Yes | KPC2 |
| ARLG-3629-P | ARLG | Yes | KPC2 |
| ARLG-3630-P | ARLG | Yes | KPC2 |
| ARLG-3631-P | ARLG | No | N/A |
| ARLG-3633-P | ARLG | Yes | KPC2 |
| ARLG-3634-P | ARLG | Yes | KPC3 |
| ARLG-3635-P | ARLG | Yes | KPC2 |
| ARLG-3637-P | ARLG | Yes | KPC3 |
| ARLG-3638-P | ARLG | Yes | KPC2 |
| ARLG-3641-P | ARLG | Yes | KPC2 |
| ARLG-3642-P | ARLG | Yes | KPC2 |
| ARLG-3643-P | ARLG | Yes | KPC2 |
| ARLG-3645-P | ARLG | No | N/A |
| ARLG-4550-P | ARLG | Yes | KPC3 |
| ARLG-4541-P | ARLG | Yes | KPC3 |
| ARLG-4542-P | ARLG | Yes | KPC2 |
| ARLG-4552-P | ARLG | Yes | KPC3 |
| ARLG-4551-P | ARLG | Yes | KPC2 |
| ARLG-4543-P | ARLG | No | N/A |
| ARLG-4289-P | ARLG | Yes | KPC2 |
| ARLG-4546-P | ARLG | Yes | KPC2 |
| ARLG-4544-P | ARLG | Yes | KPC3 |
| ARLG-4534-P | ARLG | Yes | KPC2 |
| ARLG-4537-P | ARLG | Yes | KPC3 |
| ARLG-4228-P | ARLG | Yes | KPC3 |
| ARLG-4229-P | ARLG | Yes | KPC3 |
| ARLG-4232 | ARLG | Yes | KPC3 |
| ARLG-4234 | ARLG | Yes | KPC2 |
| ARLG-4242 | ARLG | Yes | KPC2 |
| ARLG-4244 | ARLG | No | N/A |
| ARLG-4474 | ARLG | Yes | KPC3 |
| ARLG-4272 | ARLG | No | N/A |
| ARLG-4273 | ARLG | Yes | KPC2 |
| ARLG-4193 | ARLG | Yes | KPC2 |
| ARLG-4196 | ARLG | No | N/A |
| ARLG-4557 | ARLG | Yes | KPC3 |
| ARLG-4382 | ARLG | Yes | KPC2 |
| ARLG-4506 | ARLG | No | N/A |
| ARLG-4445 | ARLG | No | N/A |
| ARLG-4513 | ARLG | No | N/A |
| ARLG-4514 | ARLG | Yes | KPC3 |
| ARLG-4530 | ARLG | Yes | KPC3 |
| ARLG-4211 | ARLG | No | N/A |
| ARLG-3451-P | ARLG | No | N/A |
| ARLG-3484-P | ARLG | No | N/A |
| ARLG-4419-P | ARLG | Yes | KPC2 |
| ARLG-4340-P | ARLG | Yes | KPC8 |
| ARLG-4484-P | ARLG | Yes | KPC2 |
| ARLG-4430-P | ARLG | Yes | KPC2 |
| ARLG-4413-P | ARLG | No | N/A |
| ARLG-4489-P | ARLG | Yes | KPC2 |
| ARLG-4400-P | ARLG | Yes | KPC2 |
| ARLG-4172-P | ARLG | No | N/A |
| ARLG-3226 | ARLG | Yes | KPC3 |
| ARLG-3255 | ARLG | Yes | KPC2 |
| ARLG-3285 | ARLG | No | N/A |
| ARLG-3321 | ARLG | Yes | KPC2 |
| ARLG-3343 | ARLG | Yes | KPC3 |
| ARLG-3355 | ARLG | No | N/A |
| ARLG-3445 | ARLG | No | N/A |
| ARLG-4609 | ARLG | No | N/A |
| ARLG-4657 | ARLG | Yes | KPC3 |
| ARLG-4703 | ARLG | Yes | KPC2 |
| ARLG-4704 | ARLG | Yes | KPC3 |
| ARLG-4743 | ARLG | No | N/A |
| ARLG-4751 | ARLG | Yes | KPC2 |
| ARLG-4764 | ARLG | No | N/A |
| ARLG-4777 | ARLG | No | N/A |
| ARLG-4810 | ARLG | Yes | KPC2 |
| ARLG-4871 | ARLG | Yes | KPC2 |
| ARLG-4906 | ARLG | No | N/A |
| ARLG-4907 | ARLG | Yes | KPC3 |
