# Supplementary material for: Prediction of KPC-producing Klebsiella pneumoniae by MALDI-TOF MS, ensemble learning, and spectral peak annotation
Source: J Clin Microbiol. 2026 Mar 30;64(5):e01466-25. doi: 10.1128/jcm.01466-25 (PMC13170361; doi:10.1128/jcm.01466-25)
Supplement: Table S2 — Detailed values for validation of Training 1 group for each individual predictive model and ensemble by pairwise and triple combinations. Data for each ensemble type are sorted by decreasing specificity. [file jcm.01466-25-s0003.docx]

**Table S2.** Detailed values for validation of Training 1 group for each individual predictive model and ensemble by pairwise and triple combinations. Data for each ensemble type are sorted by decreasing specificity. PPV, positive predictive value; NPV, negative predictive value; F, full spectra; 5P, five-peak matrix; PLS-DA, partial least squares-discriminant analysis; SVM, support vector machine; LGBM, light-gradient boosting machine; RF, random forest.

| **Models** | **Sensitivity** | **Specificity** | **PPV** | **NPV** | **Proportion** | **Youden’s index** | **Balanced Accuracy** | **F1 score** |
| --- | --- | --- | --- | --- | --- | --- | --- | --- |
| 5P-PLS-DA | 41.33 | 93.67 | 89.01 | 56.27 | 100 | 0.35 | 67.5 | 0.5644903 |
| 5P-SVM | 67.35 | 76.58 | 78.11 | 65.41 | 100 | 0.4393 | 71.965 | 0.7233203 |
| 5P-LGBM | 49.49 | 89.24 | 85.09 | 58.75 | 100 | 0.3873 | 69.365 | 0.6258143 |
| 5P-RF | 54.08 | 91.14 | 88.33 | 61.54 | 100 | 0.4522 | 72.61 | 0.6708639 |
| F-PLS-DA | 3.06 | 100 | 100 | 45.4 | 100 | 0.0306 | 51.53 | 0.0593829 |
| F-LGBM | 75 | 56.33 | 68.06 | 64.49 | 100 | 0.3133 | 65.665 | 0.7136167 |
| F-RF | 74.49 | 53.16 | 66.36 | 62.69 | 100 | 0.2765 | 63.825 | 0.7019036 |
| F-SVM | 88.27 | 8.23 | 54.4 | 36.11 | 100 | -0.035 | 48.25 | 0.6731461 |
| F-PLS-DA + F-SVM | 20.69 | 100 | 100 | 36.11 | 11.86 | 0.2069 | 60.345 | 0.3428619 |
| F-PLS-DA + F-LGBM | 10.91 | 100 | 100 | 64.49 | 40.68 | 0.1091 | 55.455 | 0.1967361 |
| F-PLS-DA + F-RF | 10.71 | 100 | 100 | 62.69 | 39.55 | 0.1071 | 55.355 | 0.1934785 |
| F-PLS-DA + 5P-PLS-DA | 4.2 | 100 | 100 | 56.49 | 75.42 | 0.042 | 52.1 | 0.0806142 |
| F-PLS-DA + 5P-SVM | 8.57 | 100 | 100 | 65.41 | 53.95 | 0.0857 | 54.285 | 0.1578705 |
| F-PLS-DA + 5P-LGBM | 4.85 | 100 | 100 | 59 | 68.93 | 0.0485 | 52.425 | 0.0925131 |
| F-PLS-DA + 5P-RF | 5.32 | 100 | 100 | 61.8 | 67.23 | 0.0532 | 52.66 | 0.1010255 |
| 5P-PLS-DA + 5P-LGBM | 44.58 | 95.8 | 92.5 | 59.83 | 87.29 | 0.4038 | 70.19 | 0.6016414 |
| 5P-PLS-DA + 5P-RF | 47.27 | 95.27 | 91.76 | 61.84 | 88.42 | 0.4254 | 71.27 | 0.6239654 |
| F-LGBM + 5P-PLS-DA | 64.04 | 92.47 | 91.25 | 67.72 | 58.47 | 0.5651 | 78.255 | 0.7526112 |
| 5P-PLS-DA + 5P-SVM | 55.86 | 92.37 | 89.01 | 65.41 | 77.97 | 0.4823 | 74.115 | 0.6864221 |
| F-RF + 5P-PLS-DA | 63.03 | 92.05 | 91.46 | 64.8 | 58.47 | 0.5508 | 77.54 | 0.7462909 |
| 5P-LGBM + 5P-RF | 51.87 | 92.05 | 88.99 | 60.7 | 95.48 | 0.4392 | 71.96 | 0.6553899 |
| 5P-SVM + 5P-RF | 62.65 | 90.84 | 89.66 | 65.75 | 83.9 | 0.5349 | 76.745 | 0.7376008 |
| 5P-SVM + 5P-LGBM | 60.51 | 90 | 87.96 | 65.36 | 81.07 | 0.5051 | 75.255 | 0.7169744 |
| F-LGBM + 5P-RF | 71.43 | 88.66 | 89.62 | 69.35 | 64.97 | 0.6009 | 80.045 | 0.7949775 |
| F-RF + 5P-RF | 70.29 | 88.04 | 89.81 | 66.39 | 64.97 | 0.5833 | 79.165 | 0.7886002 |
| F-RF + 5P-LGBM | 67.15 | 86.02 | 87.62 | 64 | 64.97 | 0.5317 | 76.585 | 0.7603131 |
| F-LGBM + 5P-LGBM | 68.18 | 86 | 86.54 | 67.19 | 65.54 | 0.5418 | 77.09 | 0.7627065 |
| F-LGBM + 5P-SVM | 79.02 | 77.08 | 83.7 | 71.15 | 67.51 | 0.561 | 78.05 | 0.812927 |
| F-RF + 5P-SVM | 78.08 | 74.74 | 82.61 | 68.93 | 68.08 | 0.5282 | 76.41 | 0.8028115 |
| F-SVM + 5P-PLS-DA | 80.21 | 56.52 | 88.51 | 40.62 | 33.62 | 0.3673 | 68.365 | 0.8415585 |
| F-LGBM + F-RF | 78.36 | 55.56 | 69.07 | 66.96 | 86.44 | 0.3392 | 66.96 | 0.7342231 |
| F-SVM + 5P-RF | 87.39 | 48.15 | 87.39 | 48.15 | 38.98 | 0.3554 | 67.77 | 0.8739 |
| F-SVM + 5P-LGBM | 84.91 | 42.86 | 84.91 | 42.86 | 37.85 | 0.2777 | 63.885 | 0.8491 |
| F-SVM + 5P-SVM | 90.37 | 25 | 77.22 | 48 | 51.69 | 0.1537 | 57.685 | 0.8327909 |
| F-SVM + F-LGBM | 91.33 | 14.1 | 67.16 | 45.83 | 64.41 | 0.0543 | 52.715 | 0.7740202 |
| F-SVM + F-RF | 90.73 | 13.25 | 65.55 | 44 | 66.1 | 0.0398 | 51.99 | 0.7611149 |
| F-PLS-DA + F-SVM + F-LGBM | 31.58 | 100 | 100 | 45.83 | 8.47 | 0.3158 | 65.79 | 0.4800122 |
| F-PLS-DA + F-SVM + F-RF | 30 | 100 | 100 | 44 | 8.76 | 0.3 | 65 | 0.4615385 |
| F-PLS-DA + F-SVM + 5P-PLS-DA | 20.83 | 100 | 100 | 40.62 | 10.45 | 0.2083 | 60.415 | 0.3447819 |
| F-PLS-DA + F-SVM + 5P-SVM | 31.58 | 100 | 100 | 48 | 8.76 | 0.3158 | 65.79 | 0.4800122 |
| F-PLS-DA + F-SVM + 5P-LGBM | 23.81 | 100 | 100 | 42.86 | 9.32 | 0.2381 | 61.905 | 0.3846216 |
| F-PLS-DA + F-SVM + 5P-RF | 26.32 | 100 | 100 | 48.15 | 9.04 | 0.2632 | 63.16 | 0.4167194 |
| F-PLS-DA + F-LGBM + F-RF | 13.95 | 100 | 100 | 66.96 | 33.33 | 0.1395 | 56.975 | 0.2448442 |
| F-PLS-DA + F-LGBM + 5P-PLS-DA | 10.87 | 100 | 100 | 67.72 | 37.29 | 0.1087 | 55.435 | 0.1960855 |
| F-PLS-DA + F-LGBM + 5P-SVM | 16.67 | 100 | 100 | 71.15 | 31.07 | 0.1667 | 58.335 | 0.2857633 |
| F-PLS-DA + F-LGBM + 5P-LGBM | 10.64 | 100 | 100 | 67.19 | 37.57 | 0.1064 | 55.32 | 0.1923355 |
| F-PLS-DA + F-LGBM + 5P-RF | 11.63 | 100 | 100 | 69.35 | 36.44 | 0.1163 | 55.815 | 0.2083669 |
| F-PLS-DA + F-RF + 5P-PLS-DA | 10.2 | 100 | 100 | 64.8 | 36.72 | 0.102 | 55.1 | 0.185118 |
| F-PLS-DA + F-RF + 5P-SVM | 15.79 | 100 | 100 | 68.93 | 30.79 | 0.1579 | 57.895 | 0.2727351 |
| F-PLS-DA + F-RF + 5P-LGBM | 10 | 100 | 100 | 64 | 36.72 | 0.1 | 55 | 0.1818182 |
| F-PLS-DA + F-RF + 5P-RF | 10.87 | 100 | 100 | 66.39 | 35.88 | 0.1087 | 55.435 | 0.1960855 |
| F-PLS-DA + 5P-PLS-DA + 5P-SVM | 7.25 | 100 | 100 | 65.41 | 53.67 | 0.0725 | 53.625 | 0.1351981 |
| F-PLS-DA + 5P-PLS-DA + 5P-LGBM | 5.21 | 100 | 100 | 60.09 | 65.82 | 0.0521 | 52.605 | 0.09904 |
| F-PLS-DA + 5P-PLS-DA + 5P-RF | 5.49 | 100 | 100 | 62.11 | 65.54 | 0.0549 | 52.745 | 0.1040857 |
| F-PLS-DA + 5P-SVM + 5P-LGBM | 7.46 | 100 | 100 | 65.36 | 51.98 | 0.0746 | 53.73 | 0.1388424 |
| F-PLS-DA + 5P-SVM + 5P-RF | 7.46 | 100 | 100 | 65.75 | 52.54 | 0.0746 | 53.73 | 0.1388424 |
| F-PLS-DA + 5P-LGBM + 5P-RF | 5.32 | 100 | 100 | 60.96 | 65.82 | 0.0532 | 52.66 | 0.1010255 |
| 5P-PLS-DA + 5P-LGBM + 5P-RF | 45.96 | 95.77 | 92.5 | 60.99 | 85.59 | 0.4173 | 70.865 | 0.6140835 |
| F-LGBM + 5P-PLS-DA + 5P-LGBM | 64.81 | 95.51 | 94.59 | 69.11 | 55.65 | 0.6032 | 80.16 | 0.7691817 |
| F-RF + 5P-PLS-DA + 5P-LGBM | 62.83 | 95.18 | 94.67 | 65.29 | 55.37 | 0.5801 | 79.005 | 0.7553163 |
| 5P-PLS-DA + 5P-SVM + 5P-LGBM | 54.41 | 95.12 | 92.5 | 65.36 | 73.16 | 0.4953 | 74.765 | 0.6851712 |
| F-LGBM + 5P-PLS-DA + 5P-RF | 66.67 | 94.44 | 93.51 | 70.25 | 55.93 | 0.6111 | 80.555 | 0.7784133 |
| 5P-PLS-DA + 5P-SVM + 5P-RF | 55.71 | 94.44 | 91.76 | 65.75 | 75.14 | 0.5015 | 75.075 | 0.6932867 |
| F-RF + 5P-PLS-DA + 5P-RF | 64.6 | 94.12 | 93.59 | 66.67 | 55.93 | 0.5872 | 79.36 | 0.7643864 |
| 5P-SVM + 5P-LGBM + 5P-RF | 60.51 | 92.13 | 90.48 | 65.36 | 80.23 | 0.5264 | 76.32 | 0.7252063 |
| F-LGBM + 5P-PLS-DA + 5P-SVM | 70.87 | 91.36 | 91.25 | 71.15 | 51.98 | 0.6223 | 81.115 | 0.7977902 |
| F-LGBM + F-RF + 5P-PLS-DA | 68.27 | 91.14 | 91.03 | 68.57 | 51.69 | 0.5941 | 79.705 | 0.7802408 |
| F-RF + 5P-PLS-DA + 5P-SVM | 70.09 | 91.03 | 91.46 | 68.93 | 52.26 | 0.6112 | 80.56 | 0.7936158 |
| F-RF + 5P-LGBM + 5P-RF | 69.17 | 89.89 | 91.09 | 66.12 | 62.71 | 0.5906 | 79.53 | 0.7863092 |
| F-LGBM + 5P-LGBM + 5P-RF | 70.31 | 89.47 | 90 | 69.11 | 62.99 | 0.5978 | 79.89 | 0.7894579 |
| F-LGBM + 5P-SVM + 5P-RF | 76.42 | 89.16 | 91.26 | 71.84 | 58.19 | 0.6558 | 82.79 | 0.8318332 |
| F-RF + 5P-SVM + 5P-RF | 74.8 | 88.75 | 91.35 | 68.93 | 58.47 | 0.6355 | 81.775 | 0.8225074 |
| F-LGBM + F-RF + 5P-RF | 74.8 | 87.95 | 90.2 | 70.19 | 58.19 | 0.6275 | 81.375 | 0.8178133 |
| F-RF + 5P-SVM + 5P-LGBM | 73.77 | 87.5 | 90 | 68.63 | 57.06 | 0.6127 | 80.635 | 0.8108078 |
| F-LGBM + 5P-SVM + 5P-LGBM | 75.42 | 86.9 | 89 | 71.57 | 57.06 | 0.6232 | 81.16 | 0.8164919 |
| F-LGBM + F-RF + 5P-LGBM | 71.31 | 84.71 | 87 | 67.29 | 58.47 | 0.5602 | 78.01 | 0.7837749 |
| F-LGBM + F-RF + 5P-SVM | 80.3 | 75 | 83.46 | 70.79 | 61.02 | 0.553 | 77.65 | 0.8184951 |
| F-SVM + 5P-PLS-DA + 5P-LGBM | 82.56 | 66.67 | 92.21 | 44.44 | 29.38 | 0.4923 | 74.615 | 0.8711859 |
| F-SVM + 5P-PLS-DA + 5P-RF | 84.09 | 65 | 91.36 | 48.15 | 30.51 | 0.4909 | 74.545 | 0.8757438 |
| F-SVM + F-LGBM + 5P-PLS-DA | 85.37 | 61.11 | 90.91 | 47.83 | 28.25 | 0.4648 | 73.24 | 0.8805295 |
| F-SVM + F-RF + 5P-PLS-DA | 84.71 | 61.11 | 91.14 | 45.83 | 29.1 | 0.4582 | 72.91 | 0.8780744 |
| F-SVM + 5P-PLS-DA + 5P-SVM | 85.56 | 54.55 | 88.51 | 48 | 31.64 | 0.4011 | 70.055 | 0.8701 |
| F-SVM + F-LGBM + 5P-RF | 88.89 | 50 | 88.89 | 50 | 34.18 | 0.3889 | 69.445 | 0.8889 |
| F-SVM + F-RF + 5P-RF | 88.24 | 50 | 89.11 | 47.83 | 35.03 | 0.3824 | 69.12 | 0.8867287 |
| F-SVM + 5P-SVM + 5P-RF | 87.96 | 50 | 88.79 | 48 | 37.29 | 0.3796 | 68.98 | 0.8837305 |
| F-SVM + 5P-LGBM + 5P-RF | 86.54 | 50 | 88.24 | 46.15 | 36.16 | 0.3654 | 68.27 | 0.8738173 |
| F-SVM + 5P-SVM + 5P-LGBM | 87.13 | 45.83 | 87.13 | 45.83 | 35.31 | 0.3296 | 66.48 | 0.8713 |
| F-SVM + F-RF + 5P-LGBM | 85.86 | 43.48 | 86.73 | 41.67 | 34.46 | 0.2934 | 64.67 | 0.8629281 |
| F-SVM + F-LGBM + 5P-LGBM | 86.46 | 41.67 | 85.57 | 43.48 | 33.9 | 0.2813 | 64.065 | 0.860127 |
| F-SVM + F-LGBM + 5P-SVM | 91.38 | 31.25 | 82.81 | 50 | 41.81 | 0.2263 | 61.315 | 0.8688418 |
| F-SVM + F-RF + 5P-SVM | 90.68 | 29.41 | 81.68 | 47.62 | 42.94 | 0.2009 | 60.045 | 0.8594503 |
| F-SVM + F-LGBM + F-RF | 91.3 | 14.49 | 68.11 | 45.45 | 58.47 | 0.0579 | 52.895 | 0.7801823 |
