# Supplementary material for: Prediction of KPC-producing Klebsiella pneumoniae by MALDI-TOF MS, ensemble learning, and spectral peak annotation
Source: J Clin Microbiol. 2026 Mar 30;64(5):e01466-25. doi: 10.1128/jcm.01466-25 (PMC13170361; doi:10.1128/jcm.01466-25)
Supplement: Table S3 — Detailed values for validation of Training 2 group for each individual predictive model and ensemble by pairwise and triple combinations. Data for each ensemble type are sorted by decreasing specificity. [file jcm.01466-25-s0004.docx]

**Table S3.** Detailed values for validation of Training 2 group for each individual predictive model and ensemble by pairwise and triple combinations. Data for each ensemble type are sorted by decreasing specificity. PPV, positive predictive value; NPV, negative predictive value; F, F-spectra; 5P, five-peak matrix; PLS-DA-DA, partial least squares-discriminant analysis; SVM, support vector machine; LLGBM, light-gradient boosting machine; RF, random forest.

| **Models** | **Sensitivity** | **Specificity** | **PPV** | **NPV** | **Proportion** | **Youden’s index** | **Balanced Accuracy** | **F1 score** |
| --- | --- | --- | --- | --- | --- | --- | --- | --- |
| 5P-LGBM | 49.46 | 93.92 | 91.09 | 59.66 | 100 | 0.4338 | 71.69 | 0.641097 |
| F-LGBM | 51.61 | 89.86 | 86.49 | 59.64 | 100 | 0.4147 | 70.735 | 0.646452 |
| 5P-RF | 63.44 | 83.78 | 83.1 | 64.58 | 100 | 0.4722 | 73.61 | 0.719512 |
| 5P-SVM | 65.59 | 79.73 | 80.26 | 64.84 | 100 | 0.4532 | 72.66 | 0.721872 |
| 5P-PLS-DA | 48.92 | 92.57 | 89.22 | 59.05 | 100 | 0.4149 | 70.745 | 0.631916 |
| F-SVM | 51.08 | 86.49 | 82.61 | 58.45 | 100 | 0.3757 | 68.785 | 0.631269 |
| F-PLS-DA | 61.83 | 76.35 | 76.67 | 61.41 | 100 | 0.3818 | 69.09 | 0.68455 |
| F-RF | 58.6 | 81.08 | 79.56 | 60.91 | 100 | 0.3968 | 69.84 | 0.674901 |
| F-LGBM + 5P-LGBM | 50.66 | 99.21 | 98.72 | 62.5 | 83.23 | 0.4987 | 74.935 | 0.669588 |
| F-SVM + 5P-LGBM | 50.38 | 98.37 | 97.1 | 64.71 | 76.65 | 0.4875 | 74.375 | 0.663398 |
| F-SVM + 5P-PLS-DA | 50 | 98.35 | 97.3 | 62.3 | 79.34 | 0.4835 | 74.175 | 0.660557 |
| F-RF + 5P-LGBM | 55.56 | 98.26 | 97.4 | 65.32 | 74.85 | 0.5382 | 76.91 | 0.707576 |
| F-PLS-DA + 5P-LGBM | 58.14 | 98.15 | 97.4 | 66.25 | 70.96 | 0.5629 | 78.145 | 0.728152 |
| 5P-PLS-DA + 5P-LGBM | 49.09 | 97.76 | 96.43 | 60.93 | 89.52 | 0.4685 | 73.425 | 0.650598 |
| F-PLS-DA + 5P-PLS-DA | 57.35 | 97.22 | 96.3 | 64.42 | 73.05 | 0.5457 | 77.285 | 0.718881 |
| F-SVM + F-LGBM | 51.85 | 96.69 | 94.59 | 64.29 | 76.65 | 0.4854 | 74.27 | 0.66983 |
| F-RF + 5P-PLS-DA | 55.22 | 96.58 | 94.87 | 65.32 | 75.15 | 0.518 | 75.9 | 0.698077 |
| F-LGBM + 5P-PLS-DA | 50.33 | 96.21 | 93.83 | 62.87 | 84.73 | 0.4654 | 73.27 | 0.65517 |
| F-LGBM + F-RF | 57.14 | 95.65 | 93.83 | 65.87 | 74.25 | 0.5279 | 76.395 | 0.710266 |
| F-SVM + 5P-RF | 60.15 | 95.61 | 94.12 | 67.28 | 73.95 | 0.5576 | 77.88 | 0.733949 |
| F-PLS-DA + F-LGBM | 59.26 | 95.37 | 94.12 | 65.19 | 72.75 | 0.5463 | 77.315 | 0.727285 |
| 5P-LGBM + 5P-RF | 58.82 | 95.28 | 93.02 | 68.36 | 78.74 | 0.541 | 77.05 | 0.720685 |
| F-LGBM + 5P-RF | 60.14 | 95.04 | 93.26 | 67.65 | 77.54 | 0.5518 | 77.59 | 0.731246 |
| 5P-SVM + 5P-LGBM | 58.97 | 93.6 | 92 | 64.64 | 84.13 | 0.5257 | 76.285 | 0.718718 |
| F-LGBM + 5P-SVM | 60.96 | 93.28 | 91.75 | 66.07 | 79.34 | 0.5424 | 77.12 | 0.73251 |
| F-PLS-DA + 5P-RF | 67.15 | 93.2 | 92.93 | 68.09 | 71.86 | 0.6035 | 80.175 | 0.779641 |
| F-SVM + F-RF | 56.92 | 93.1 | 90.24 | 65.85 | 73.65 | 0.5002 | 75.01 | 0.698078 |
| 5P-PLS-DA + 5P-RF | 58.27 | 92.48 | 89.01 | 67.96 | 81.44 | 0.5075 | 75.375 | 0.70432 |
| F-SVM + 5P-SVM | 61.48 | 91.53 | 89.25 | 67.5 | 75.75 | 0.5301 | 76.505 | 0.728069 |
| 5P-PLS-DA + 5P-SVM | 58.71 | 91.47 | 89.22 | 64.84 | 85.03 | 0.5018 | 75.09 | 0.708187 |
| F-RF + 5P-SVM | 66.42 | 90.18 | 89.22 | 68.71 | 74.55 | 0.566 | 78.3 | 0.7615 |
| F-RF + 5P-RF | 61.99 | 90 | 89.83 | 62.43 | 87.13 | 0.5199 | 75.995 | 0.733574 |
| F-PLS-DA + 5P-SVM | 68.89 | 89.52 | 89.42 | 69.12 | 71.86 | 0.5841 | 79.205 | 0.778238 |
| F-PLS-DA + F-RF | 63.57 | 88.29 | 87.25 | 65.77 | 75.15 | 0.5186 | 75.93 | 0.73551 |
| F-PLS-DA + F-SVM | 58.11 | 86.61 | 83.5 | 63.95 | 82.34 | 0.4472 | 72.36 | 0.685289 |
| 5P-SVM + 5P-RF | 68 | 84.56 | 82.93 | 70.55 | 85.63 | 0.5256 | 76.28 | 0.747266 |
| F-PLS-DA + F-LGBM + 5P-LGBM | 57.39 | 100 | 100 | 66.44 | 63.47 | 0.5739 | 78.695 | 0.729271 |
| F-SVM + F-LGBM + 5P-PLS-DA | 50 | 100 | 100 | 64.94 | 70.36 | 0.5 | 75 | 0.666667 |
| F-SVM + F-LGBM + 5P-LGBM | 49.57 | 100 | 100 | 65.29 | 68.26 | 0.4957 | 74.785 | 0.662834 |
| F-LGBM + F-RF + 5P-LGBM | 57.26 | 100 | 100 | 67.53 | 66.17 | 0.5726 | 78.63 | 0.728221 |
| F-LGBM + 5P-PLS-DA + 5P-LGBM | 51.06 | 100 | 100 | 63.87 | 78.74 | 0.5106 | 75.53 | 0.676023 |
| F-LGBM + 5P-LGBM + 5P-RF | 59.17 | 100 | 100 | 69.75 | 69.76 | 0.5917 | 79.585 | 0.743482 |
| F-RF + 5P-PLS-DA + 5P-LGBM | 54.03 | 100 | 100 | 65.66 | 69.76 | 0.5403 | 77.015 | 0.701552 |
| F-SVM + 5P-PLS-DA + 5P-LGBM | 50 | 99.13 | 98.46 | 64.04 | 72.75 | 0.4913 | 74.565 | 0.663209 |
| F-LGBM + 5P-SVM + 5P-LGBM | 57.46 | 99.1 | 98.72 | 65.87 | 73.35 | 0.5656 | 78.28 | 0.726399 |
| F-SVM + F-LGBM + 5P-RF | 58.18 | 99.04 | 98.46 | 69.13 | 64.07 | 0.5722 | 78.61 | 0.73141 |
| F-SVM + F-RF + 5P-PLS-DA | 55.86 | 99.03 | 98.41 | 67.55 | 64.07 | 0.5489 | 77.445 | 0.71267 |
| F-SVM + F-RF + 5P-LGBM | 54.72 | 99.03 | 98.31 | 68 | 62.57 | 0.5375 | 76.875 | 0.703068 |
| F-SVM + F-LGBM + F-RF | 54.72 | 99.02 | 98.31 | 67.79 | 62.28 | 0.5374 | 76.87 | 0.703068 |
| F-PLS-DA + 5P-PLS-DA + 5P-LGBM | 56.56 | 99.01 | 98.57 | 65.36 | 66.77 | 0.5557 | 77.785 | 0.718767 |
| F-PLS-DA + F-LGBM + 5P-PLS-DA | 55.93 | 99 | 98.51 | 65.56 | 65.27 | 0.5493 | 77.465 | 0.713502 |
| F-PLS-DA + F-RF + 5P-PLS-DA | 61.61 | 98.94 | 98.57 | 68.38 | 61.68 | 0.6055 | 80.275 | 0.758259 |
| F-PLS-DA + F-RF + 5P-LGBM | 61.47 | 98.92 | 98.53 | 68.66 | 60.48 | 0.6039 | 80.195 | 0.75708 |
| 5P-PLS-DA + 5P-LGBM + 5P-RF | 56.69 | 98.37 | 97.3 | 68.75 | 74.85 | 0.5506 | 77.53 | 0.716402 |
| F-SVM + 5P-PLS-DA + 5P-SVM | 58.06 | 98.18 | 97.3 | 67.5 | 70.06 | 0.5624 | 78.12 | 0.727245 |
| F-SVM + 5P-PLS-DA + 5P-RF | 57.39 | 98.18 | 97.06 | 68.79 | 67.37 | 0.5557 | 77.785 | 0.721304 |
| F-SVM + 5P-SVM + 5P-LGBM | 56.3 | 98.17 | 97.1 | 67.3 | 68.26 | 0.5447 | 77.235 | 0.712742 |
| F-SVM + 5P-LGBM + 5P-RF | 56.48 | 98.15 | 96.83 | 69.28 | 64.67 | 0.5463 | 77.315 | 0.713451 |
| F-LGBM + F-RF + 5P-PLS-DA | 56.03 | 98.15 | 97.01 | 67.52 | 67.07 | 0.5418 | 77.09 | 0.710333 |
| F-RF + 5P-LGBM + 5P-RF | 58.59 | 98.13 | 97.4 | 66.46 | 70.36 | 0.5672 | 78.36 | 0.731671 |
| F-PLS-DA + F-SVM + 5P-LGBM | 55.36 | 98.1 | 96.88 | 67.32 | 64.97 | 0.5346 | 76.73 | 0.704582 |
| F-SVM + F-LGBM + 5P-SVM | 58.26 | 98.1 | 97.1 | 68.21 | 65.87 | 0.5636 | 78.18 | 0.72825 |
| F-PLS-DA + F-SVM + 5P-PLS-DA | 55.37 | 98.08 | 97.1 | 65.38 | 67.37 | 0.5345 | 76.725 | 0.705244 |
| F-LGBM + F-RF + 5P-RF | 58.59 | 98.08 | 97.4 | 65.81 | 69.46 | 0.5667 | 78.335 | 0.731671 |
| F-RF + 5P-SVM + 5P-LGBM | 61.98 | 98.04 | 97.4 | 68.49 | 66.77 | 0.6002 | 80.01 | 0.757542 |
| F-LGBM + F-RF + 5P-SVM | 63.48 | 97.98 | 97.33 | 69.78 | 64.07 | 0.6146 | 80.73 | 0.768423 |
| F-PLS-DA + 5P-SVM + 5P-LGBM | 64.1 | 97.89 | 97.4 | 68.89 | 63.47 | 0.6199 | 80.995 | 0.773169 |
| F-PLS-DA + 5P-LGBM + 5P-RF | 62.96 | 97.89 | 97.14 | 69.92 | 60.78 | 0.6085 | 80.425 | 0.764014 |
| F-PLS-DA + F-LGBM + F-RF | 62.16 | 97.85 | 97.18 | 68.42 | 61.08 | 0.6001 | 80.005 | 0.758216 |
| F-PLS-DA + F-LGBM + 5P-RF | 64.29 | 97.83 | 97.3 | 69.23 | 61.08 | 0.6212 | 81.06 | 0.774233 |
| 5P-PLS-DA + 5P-SVM + 5P-LGBM | 55.86 | 97.5 | 96.43 | 64.64 | 79.34 | 0.5336 | 76.68 | 0.707411 |
| F-PLS-DA + F-SVM + F-LGBM | 56.03 | 97.12 | 95.59 | 66.45 | 65.87 | 0.5315 | 76.575 | 0.706491 |
| F-SVM + F-RF + 5P-RF | 58.87 | 97.03 | 96.05 | 65.77 | 67.37 | 0.559 | 77.95 | 0.729985 |
| F-PLS-DA + 5P-PLS-DA + 5P-RF | 62.83 | 96.94 | 95.95 | 69.34 | 63.17 | 0.5977 | 79.885 | 0.759357 |
| F-PLS-DA + 5P-PLS-DA + 5P-SVM | 65 | 96.91 | 96.3 | 69.12 | 64.97 | 0.6191 | 80.955 | 0.776131 |
| F-PLS-DA + F-RF + 5P-RF | 66.41 | 96.77 | 96.67 | 67.16 | 67.07 | 0.6318 | 81.59 | 0.787326 |
| F-PLS-DA + F-LGBM + 5P-SVM | 65.22 | 96.74 | 96.15 | 68.99 | 61.98 | 0.6196 | 80.98 | 0.777208 |
| F-RF + 5P-PLS-DA + 5P-RF | 57.36 | 96.4 | 94.87 | 66.05 | 71.86 | 0.5376 | 76.88 | 0.714937 |
| F-RF + 5P-PLS-DA + 5P-SVM | 61.67 | 96.19 | 94.87 | 68.71 | 67.37 | 0.5786 | 78.93 | 0.747494 |
| F-SVM + F-RF + 5P-SVM | 63.89 | 95.96 | 94.52 | 70.9 | 61.98 | 0.5985 | 79.925 | 0.762437 |
| F-LGBM + 5P-PLS-DA + 5P-RF | 57.85 | 95.8 | 93.33 | 69.09 | 71.86 | 0.5365 | 76.825 | 0.714267 |
| F-LGBM + 5P-PLS-DA + 5P-SVM | 57.14 | 95.69 | 93.83 | 66.07 | 74.55 | 0.5283 | 76.415 | 0.710266 |
| F-PLS-DA + F-RF + 5P-SVM | 71.68 | 95.56 | 95.29 | 72.88 | 60.78 | 0.6724 | 83.62 | 0.818157 |
| F-SVM + 5P-SVM + 5P-RF | 64.66 | 95.45 | 93.75 | 71.92 | 67.66 | 0.6011 | 80.055 | 0.76534 |
| 5P-SVM + 5P-LGBM + 5P-RF | 62.5 | 95 | 93.02 | 70.37 | 74.25 | 0.575 | 78.75 | 0.747653 |
| F-PLS-DA + F-SVM + 5P-RF | 63.79 | 94.9 | 93.67 | 68.89 | 64.07 | 0.5869 | 79.345 | 0.75895 |
| F-LGBM + 5P-SVM + 5P-RF | 64.52 | 94.74 | 93.02 | 71.05 | 71.26 | 0.5926 | 79.63 | 0.761921 |
| F-PLS-DA + 5P-SVM + 5P-RF | 72.03 | 92.86 | 92.39 | 73.39 | 64.67 | 0.6489 | 82.445 | 0.809494 |
| F-PLS-DA + F-SVM + F-RF | 60.34 | 92.23 | 89.74 | 67.38 | 65.57 | 0.5257 | 76.285 | 0.721603 |
| 5P-PLS-DA + 5P-SVM + 5P-RF | 62.79 | 92 | 89.01 | 70.55 | 76.05 | 0.5479 | 77.395 | 0.736356 |
| F-PLS-DA + F-SVM + 5P-SVM | 65.52 | 91.09 | 89.41 | 69.7 | 64.97 | 0.5661 | 78.305 | 0.756231 |
| F-RF + 5P-SVM + 5P-RF | 66.91 | 90.91 | 90.1 | 68.97 | 73.65 | 0.5782 | 78.91 | 0.767925 |
