# Supplementary material for: Prediction of KPC-producing Klebsiella pneumoniae by MALDI-TOF MS, ensemble learning, and spectral peak annotation
Source: J Clin Microbiol. 2026 Mar 30;64(5):e01466-25. doi: 10.1128/jcm.01466-25 (PMC13170361; doi:10.1128/jcm.01466-25)
Supplement: Table S4 — List of the 71 peaks for which a presumptive identification was obtained, including the name of the protein and accession number to Uniprot database. [file jcm.01466-25-s0005.docx]

**Table S4.** List of the 71 peaks for which a presumptive identification was obtained, including the name of the protein and accession number to Uniprot database.

| **Peak (m/z)** | **Protein** | **Uniprot access** |
| --- | --- | --- |
| 2182 | RL36 [M+2H]^2+^ | A0A2W0JL91 |
| 2690 | RL34 [M+2H]^2+^ | A6TG05 |
| 3076 | Uncharacterized protein YmdF [M+2H]^2+^ | NA |
| 3144 | RL33 [M+CH3]^2+^ | A6TFM7 |
| 3149 | RL32 [M+2H]^2+^ | A6T7E9 |
| 3191 | RL30 [M+2H]^2+^ | A6TEV4 |
| 3579 | RL35 [M+2H]^2+^ | B5XQC8 |
| 3622 | RL29 [M+2H]^2+^ | A6TEW4 |
| 3715 | Uncharacterized protein YnfD [M+2H]^2+^ | A0A1Y0PY79 |
| 3863 | Protein AaeX [M+2H]^2+^ | B5XSP7 |
| 3871 | RL31 [M+2H]^2+^ | A6TGC6 |
| 4154 | UPF0337 protein YjbJ [M+2H]^2+^ | A0A0H3GLX0 |
| 4177 | Protein SlyX [M+2H]^2+^ | A6TEY6 |
| 4184 | RS21 [M+2H]^2+^ | A6TE47 |
| 4196 | Cytochrome bd-I ubiquinol oxidase subunit X [M+H]^+^ | A0A080SLJ1 |
| 4365 | RL36 [M+H]^+^ | A0A2W0JL91 |
| 4438 | RL28 [M+2H]^2+^ | A6TFM8 |
| 4450 | Osmotically-inducible protein Y [M+2H]^2+^ | A0A927DFJ6 |
| 4496 | RL27 [M+2H]^2+^ | A6TEK4 |
| 4510 | Osmotically-inducible lipoprotein B [M+H]^+^ | W1DG62 |
| 4570 | DNA-binding protein HU-beta [M+2H]^2+^ | A6T5I3 |
| 4647 | Nickel/cobalt homeostasis protein RcnB [M+2H]^2+^ | A0A0J4QJ68 |
| 4739 | DNA-binding protein HU-alpha [M+2H]^2+^ | A6TGQ7 |
| 4750 | Protein Rof [M+2H]^2+^ | B5Y1I2 |
| 4760 | Uncharacterized protein YdbL [M+2H]^2+^ | A0A1Y0Q0V6 |
| 4768 | RS17 [M+2H]^2+^ | A0A5C2LIK1 |
| 4927 | Ethanolamine utilization protein eutN [M+2H]^2+^ | B5XVQ0 |
| 5039 | RS15 [M+2H]^2+^ | A6TEI4 |
| 5142 | RS19 [M+2H]^2+^ | A6TEW8 |
| 5280 | Stationary-phase-induced ribosome-associated protein [M+H]^+^ | W1DGS8 |
| 5379 | RL34 [M+H]^+^ | A6TG05 |
| 5555 | pKpQIL_p019 [M+2H]^2+^ | A0A844PX96 |
| 5612 | Integration host factor subunit alpha [M+2H]^2+^ | A6TAI1 |
| 5723 | RS14 [M+2H]^2+^ | B5XNA6 |
| 5760 | Uncharacterized protein YhdV [M+H]^+^ | A6TET2 |
| 5940 | Heat shock protein HspQ [M+2H]^2+^ | A6T765 |
| 6152 | Uncharacterized protein YmdF [M+H]^+^ | NA |
| 6288 | RL33 [M+CH3]^+^ | A6TFM7 |
| 6300 | RL32 [M+H]^+^ | A6T7E9 |
| 6383 | RL30 [M+H]^+^ | A6TEV4 |
| 7159 | RL35 [M+H]^+^ | B5XQC8 |
| 7244 | RL29 [M+H]^+^ | A6TEW4 |
| 7431 | Uncharacterized protein YnfD [M+H]^+^ | A0A1Y0PY79 |
| 7726 | Protein AaeX [M+H]^+^ | B5XSP7 |
| 7743 | RL31 [M+H]^+^ | A6TGC6 |
| 8309 | UPF0337 protein YjbJ [M+H]^+^ | A0A0H3GLX0 |
| 8355 | Protein SlyX [M+H]^+^ | A6TEY6 |
| 8368 | RS21 [M+H]^+^ | A6TE47 |
| 8856 | RS18 [M+H]^+^ | A6THB3 |
| 8876 | RL28 [M+H]^+^ | A6TFM8 |
| 8898 | Osmotically-inducible protein Y [M+H]^+^ | A0A927DFJ6 |
| 8993 | RL27 [M+H]^+^ | A6TEK4 |
| 9138 | DNA-binding protein HU-beta [M+H]^+^ | A6T5I3 |
| 9290 | Nickel/cobalt homeostasis protein RcnB [M+H]^+^ | A0A0J4QJ68 |
| 9478 | DNA-binding protein HU-alpha [M+H]^+^ | A6TGQ7 |
| 9500 | Protein Rof [M+H]^+^ | B5Y1I2 |
| 9521 | Uncharacterized protein YdbL [M+H]^+^ | A0A1Y0Q0V6 |
| 9540 | RS17 [M+H]^+^ | A0A5C2LIK1 |
| 9849 | Ethanolamine utilization protein eutN [M+H]^+^ | B5XVQ0 |
| 10079 | RS15 [M+H]^+^ | A6TEI4 |
| 10285 | RS19 [M+H]^+^ | A6TEW8 |
| 10580 | RL25 [M+H]^+^ | A6TBR5 |
| 10737 | Uncharacterized protein YoaC [M+H]^+^ | A0A087FPS2 |
| 10760 | Ribosome hibernation promoting factor [M+H]^+^ | A6TEM2 |
| 10873 | RNA-binding protein Hfq [M+H]^+^ | A6TH87 |
| 10885 | Uncharacterized protein YqjD [M+H]^+^ | W1DJS2 |
| 11109 | pKpQIL_p019 [M+H]^+^ | A0A844PX96 |
| 11224 | Integration host factor subunit alpha [M+H]^+^ | A6TAI1 |
| 11447 | RS14 [M+H]^+^ | B5XNA6 |
| 11880 | Heat shock protein HspQ [M+H]^+^ | A6T765 |
| 12772 | Glutaredoxin 4 [M+H]^+^ | A0A0J2J8V6 |
